# Leveraging Artificial Intelligence and Machine Learning for Characterizing Protein Corona, Nanobiological Interactions, and Advancing Drug Discovery

**DOI:** 10.3390/bioengineering12030312

**Published:** 2025-03-18

**Authors:** Turkan Kopac

**Affiliations:** Department of Chemistry, Zonguldak Bülent Ecevit University, 67100 Zonguldak, Türkiye; turkan.kopac@beun.edu.tr

**Keywords:** artificial intelligence, drug discovery, machine learning, nanomedicines, nanobio interactions, protein corona, protein–protein interactions

## Abstract

Proteins are essential for all living organisms, playing key roles in biochemical reactions, structural support, signal transduction, and gene regulation. Their importance in biomedical research is highlighted by their role as drug targets in various diseases. The interactions between proteins and nanoparticles (NPs), including the protein corona’s formation, significantly affect NP behavior, biodistribution, cellular uptake, and toxicity. Comprehending these interactions is pivotal for advancing the design of NPs to augment their efficacy and safety in biomedical applications. While traditional nanomedicine design relies heavily on experimental work, the use of data science and machine learning (ML) is on the rise to predict the synthesis and behavior of nanomaterials (NMs). Nanoinformatics combines computational simulations with laboratory studies, assessing risks and revealing complex nanobio interactions. Recent advancements in artificial intelligence (AI) and ML are enhancing the characterization of the protein corona and improving drug discovery. This review discusses the advantages and limitations of these approaches and stresses the importance of comprehensive datasets for better model accuracy. Future developments may include advanced deep-learning models and multimodal data integration to enhance protein function prediction. Overall, systematic research and advanced computational tools are vital for improving therapeutic outcomes and ensuring the safe use of NMs in medicine.

## 1. Introduction

### 1.1. Understanding Protein Functions

Proteins are essential macromolecules vital for the survival and functioning of all living organisms. They perform numerous critical roles, including participating in biochemical reactions, providing structural integrity to cells and tissues, facilitating communication within and between cells, and regulating gene expression. Proteins are involved in a variety of functions, such as offering structural support, enabling biochemical reactions, managing gene expression, and facilitating signal transduction. The diverse functions of proteins in various biological processes underscore their importance in maintaining cellular balance and promoting overall health in organisms [1,2,3]. Proteins can catalyze chemical reactions, driving billions of biochemical processes, and often form larger macromolecular complexes. The structures and functional roles of proteins remain a significant focus of ongoing research [1,2]. Understanding the functions of proteins is a critical step in comprehending biological systems and influencing biological processes, which are essential in biomedical research and the development of biotechnologies. Moreover, proteins frequently serve as targets in drug discovery [4,5,6,7,8,9] because of their involvement in various diseases. Gaining insights into protein functions can aid in the development of targeted therapies [10].

The traditional methods employed to experimentally examine the structures of protein complexes include electron microscopy, X-ray crystallography, Raman spectroscopy, and NMR spectroscopy. The functions of these proteins can be assessed through techniques such as enzymatic analysis and biochemical assays [10,11,12,13]. However, these experimental approaches for elucidating protein function are often costly, time-consuming, and effortful, and they can only be employed for a restricted number of proteins. Consequently, given the current pace of structure determination, it may take a minimum of twenty years to obtain a comprehensive library of protein complex structures. Thus, the ability to predict protein functions computationally is essential for addressing the need for functional information on the majority of proteins, presenting a significant challenge within the field of bioinformatics [10]. At present, many protein sequences, totaling hundreds of millions, have been produced as a result of various genome and transcriptome sequencing initiatives. Nonetheless, the protein functions of less than 1% of these sequences have been confirmed through experimental data. This reveals a notable disparity between the identified protein sequences and their corresponding functions. As a result, it is essential to develop sophisticated computational techniques capable of reliably predicting protein functions, akin to the advancements achieved in deep learning (DL) for protein structure prediction and determination [10,14,15,16,17,18]. This situation emphasizes the pressing need to develop effective computational techniques capable of reliably predicting the structures of protein complexes, particularly when the structures of homologous proteins are unavailable [19].

### 1.2. Exploring Nanobio Interactions: The Essential Role of Nanoinformatics

Proteins seldom function independently of the densely packed environment of a cell [20]. Their biological activities are closely tied to the partners with whom they interact [21]. Proteins have the ability to interact with various molecules. These interaction partners include ions, small organic compounds, membrane lipids, nucleic acids, small peptides, and other proteins, leading to the formation of both homo and heterocomplexes. Within the densely packed environment of the cell, proteins have evolved to achieve and sustain the efficiency and binding specificity essential for their function. The structural characteristics at the binding interfaces largely determine the physical interactions among proteins [1,2,22,23,24,25,26]. Research has shown that the binding interfaces in distinct protein complexes are highly similar. The structural characteristics of various binding interfaces can be effectively captured through AI. ML approaches hold significant potential for discovering and predicting the conformations of previously uncharacterized protein–protein interactions (PPIs) [19].

NMs have rapidly advanced across various disciplines; however, much of the research on NMs and nanotechnology relies on expensive experimental methods or complex calculations, such as the density functional theory (DFT) [27]. The properties of NPs—including shape, size, and surface chemistry—play a crucial role in determining their functions [28,29,30]. For NPs to be effectively utilized in theranostics, they must be engineered with precisely controlled characteristics, which requires the use of multiple reagents and interconnected experimental conditions [31,32,33]. Currently, nanomedicine research tends to focus on a limited range of NMs. As the demand for NMs to address biomedical challenges increases, the discovery of new materials has become a key area of interest within nanomedicine research. Scientists are actively investigating innovative material entities to broaden the options for nanoformulations that can address complex delivery challenges, such as stability in both physical and biological contexts, the immune response, and clearance by the reticuloendothelial system. Centralized platforms that offer guidance for nanoformulations could significantly accelerate research initiatives [34]. Carbon-based NMs, including graphene, carbon nanotubes (CNTs), and fullerenes, have garnered considerable attention because of their remarkable properties and potential applications across various fields, particularly in biomedicine. Comprehending how they interact with plasma proteins is essential for evaluating their cytotoxicity and biocompatibility with biological systems [12,35,36] and their prospective use in biomedical applications. Blood plasma proteins are generally categorized into three main groups: albumins, globulins, and fibrinogen, with albumins being the most prevalent [37].

**Figure 1:**
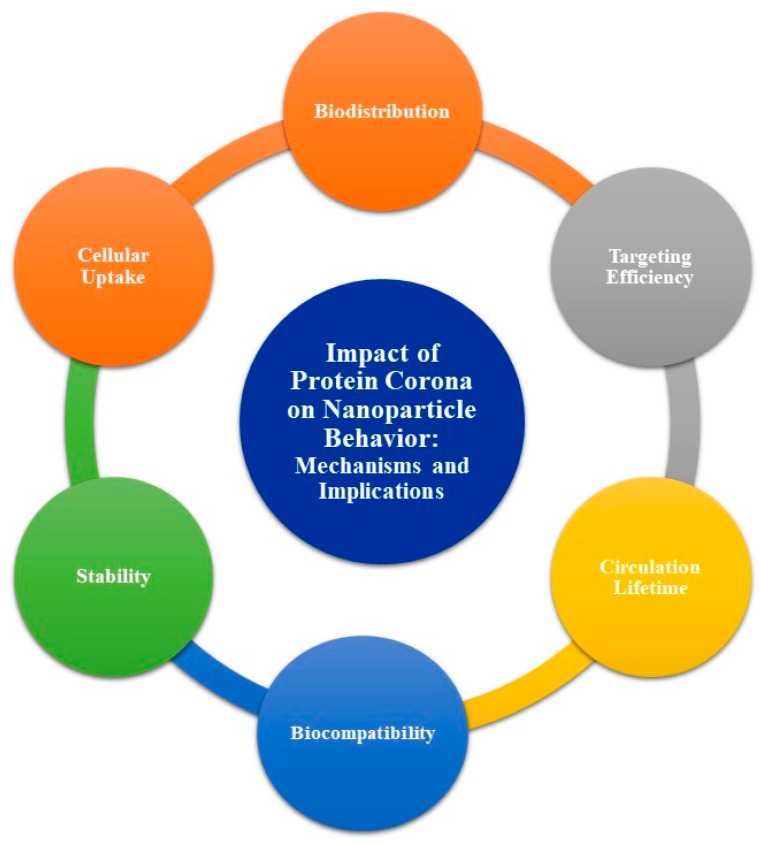
Impact of protein corona on nanoparticle behavior: Mechanisms and implications.

The protein corona is a layer of biomolecules, primarily composed of proteins, that form around NPs when they enter a biological environment. This layer emerges as a result of protein adsorption onto the NP surface. The formation of the protein corona significantly influences the behavior, biodistribution, cellular uptake, and toxicity of NPs within biological systems [12,38,39,40,41,42,43]. Understanding the protein corona is essential for the development of effective NP-based drug delivery systems and other biomedical applications. The key factors that influence protein corona formation include the following (Table 1): NP characteristics, the physicochemical properties of the NPs, the environmental conditions, the protein binding affinities, the duration of NP exposure to the biological environment, the Vroman effect, and the protein concentration. These factors collectively determine the nature, composition, and biological implications of the protein corona surrounding NPs. The protein corona affects NP behavior in several significant ways, as shown in Figure 1 [12], which depicts the mechanisms and implications of this effect.

- *Biodistribution*: The protein corona can alter the distribution of NPs within the body, influencing their travel and accumulation in various tissues and organs.
- *Cellular uptake*: The composition and structure of the protein corona can affect how cells recognize and internalize NPs, impacting their efficacy in drug delivery and other therapeutic applications.
- *Stability*: The protein corona may enhance or reduce the stability of NPs in biological environments, affecting their aggregation and solubility.
- *Biocompatibility*: The presence of a protein corona can modify the immune response to NPs, potentially increasing or decreasing their immunogenicity and toxicity.
- *Circulation lifetime*: The protein corona may influence the circulation time of NPs in the bloodstream, affecting their ability to reach target sites before being cleared from the body.
- *Targeting efficiency*: The protein corona can mask or alter the surface properties of NPs, potentially hindering their ability to bind to specific target cells or tissues.

Understanding the effects of protein adsorption on NP surfaces is essential for optimizing their design and application in biomedical fields. The interaction between proteins and NP surfaces significantly impacts several critical factors, including surface properties, stability, biocompatibility, cellular uptake, circulation time, targeting efficiency, functionality, and toxicity (Table 2). Therefore, a thorough comprehension of protein adsorption dynamics is vital for advancing the efficacy of NPs in various biomedical domains. Numerous studies have focused on characterizing protein adsorption properties across a variety of surfaces [26,30,43,44,45,46,47,48]. Among these surfaces, carbon nanotubes (CNTs) have garnered considerable attention because of their unique physicochemical properties and promising applications in biomedicine [49,50,51,52]. Research indicates that the adsorption of bovine serum albumin (BSA) is markedly influenced by several parameters, including pH, temperature, and the intrinsic surface characteristics of the materials [49,53,54]. It has been demonstrated that surface modifications can considerably affect both the adsorption behavior and the conformation of adsorbed proteins [55]. For example, BSA adsorption on multiwalled carbon nanotubes (MWCNTs) tends to increase with increasing temperature and adsorbent dosage, whereas pH has the opposite effect. Notably, the adsorption capacity is greater at lower pH values, suggesting the dominance of strong electrostatic interactions between BSA and the MWCNTs [49]. Similarly, BSA adsorption on doublewalled carbon nanotubes (DWCNTs) exhibited optimal capacity at pH 4 and 40 °C, characterized by electrostatic attractions between positively charged protein molecules and negatively charged CNT surfaces [52]. These findings exemplify the significant diversity of interactions between NPs and proteins, emphasizing the necessity for comprehending these interactions to enhance NP design and functionality in biomedical applications. The versatility of CNTs in adsorbing various proteins offers considerable potential for applications in drug delivery, biosensing, and other biomedical fields. A comprehensive understanding of the factors influencing protein adsorption—such as pH, temperature, and surface functionalization—is crucial for optimizing the performance of CNTs in these applications.

The design of nanomedicines often relies on trial and error, necessitating extensive benchwork to optimize formulations and properties. To expedite progress, data science and ML are increasingly leveraged to predict the synthesis and biological behavior of NMs. Nanoinformatics has emerged as a significant area within nanobiotechnology, playing a significant role in revealing complex molecular interactions at the nanobio interface. This field aids in risk assessment related to NMs and offers new insights into their theranostic potential. It incorporates computational simulations to complement laboratory studies and enhance the understanding of NMs and their biological interactions. Computational simulations can predict the behavior and properties of NMs, which helps in designing experiments and interpreting experimental data. This complementary approach allows for a more comprehensive assessment of risks and the revelation of complex nanobio interactions, ultimately accelerating the development of safe and effective nanomedicines. As scientific disciplines become more data-driven, nanoinformatics integrates computer science, information technology, nanotechnology, and medicine to facilitate the discovery of NMs. The focus lies in informatics techniques for analyzing the structural and physicochemical properties of NMs, thereby accelerating their clinical application [34,37,56,57]. Nevertheless, effective data curation methods are essential for managing large datasets and often operating independently from AI applications. This disconnect leads to the use of small datasets with limited translational value. Moreover, the lack of standardized reporting metrics hinders comparability, and access to centralized databases remains a challenge for many researchers [34]. 

AI approaches, such as ML and DL, have the potential to significantly accelerate the development of NM preparation protocols and facilitate the discovery of new NMs by predicting nanobio interactions. DL models enhance protein function prediction by integrating multiple data modalities, such as sequences, structures, and interactions, for a holistic understanding of protein roles. These models leverage evolutionary information to improve accuracy and can be fine-tuned for specific prediction tasks using large language models for proteins (LLMPs). Advanced neural architectures, such as convolutional and recurrent networks, capture complex patterns, while few-shot and zero-shot learning enhances predictions for rare or novel proteins. Additionally, attention mechanisms and explainable AI improve interpretability, providing valuable insights. These advancements promise greater accuracy and reliability in protein function predictions. Nevertheless, their efficiency is presently limited by the absence of appropriate nanodescriptors and labeling techniques [58,59,60,61,62]. While biological data have improved, and powerful ML tools are now available for tasks such as bioimage analysis and protein structure prediction [2,63], several challenges persist. These include knowledge gaps, the need for better interpretability of ML algorithms, limited database accuracy, and difficulties in nanopattern recognition, all of which adversely affect NM research [27]. AI is profoundly transforming various industries, particularly bioinformatics, which focuses on the analysis of biological data through sophisticated methods and tools. Recent advancements in AI have enhanced the computational techniques for predicting interactions between proteins and DNA/RNA, marking a transition from traditional ML approaches to more advanced DL methodologies [64].

This review outlines recent advancements in ML and DL methodologies used for protein corona characterization, nanobio interactions, nanomedicine development, drug discovery processes, and protein–protein interactions. It critically evaluates the advantages and limitations of these approaches, their diverse applications across various fields, and potential future trends in this rapidly evolving domain. The paper provides a comprehensive examination of recent advancements in AI and ML techniques applied to different aspects of nanobiotechnology and drug discovery. The novelty of this paper lies in its systematic exploration of how AI and ML can be utilized to

1. Characterize protein corona: The paper illustrates the use of ML models to predict the relative protein abundance (RPA) in the protein corona, which reduces the reliance on traditional experimental techniques and offers insights for designing the protein corona.
2. Understand nanobio interactions: It emphasizes the importance of systematically investigating nanobio interactions and the role of nanoinformatics in assessing risks and revealing complex interactions at the nanobio interface.
3. Advance nanomedicine and drug discovery: The review discusses how AI and ML can enhance the design and application of NPs in areas such as nanomedicine, biosensing, and organ targeting, and improve drug discovery processes by integrating data from various omics fields.
4. Predict PPIs: The paper reviews ML and DL methods for predicting PPIs, highlighting the potential of these techniques to deepen our understanding of protein functions and interactions.
5. Address challenges and future directions: It critically examines the advantages and limitations of these approaches, emphasizing the need for comprehensive datasets, advanced learning models, and multimodal data integration to enhance model accuracy and reliability.

Overall, the paper underscores the transformative potential of AI and ML in various scientific and medical fields while acknowledging ongoing challenges and the necessity for continued progress and collaboration.

## 2. Evaluating the Literature on the Application of AI and ML Techniques for Characterizing Protein Corona, Nanobio Interactions, Nanomedicines and Drug Discovery, and Protein–Protein Interactions

In the present evaluation, the literature studies conducted have been systematically categorized into four primary domains: protein corona characterization, nanobio interactions, nanomedicines and drug discovery, as well as protein–protein interactions. A comprehensive evaluation of these studies was performed, and the key findings from this analysis are summarized in Table 3.

### 2.1. Protein Corona Characterization

The protein corona plays a crucial role in modulating the behavior and functionality of NPs in biological environments. It also significantly impacts the interactions between NPs and cancer cells, influencing their therapeutic efficacy and biodistribution. Once NPs are introduced into physiological fluids, their surface properties undergo notable alterations. This “identity change” impacts their biochemical and physical characteristics, affecting cell targeting, systemic circulation, cellular uptake, and biocompatibility. Research has demonstrated that the behavior of the protein corona varies between positively charged and negatively charged NPs. Positively charged NPs attract more proteins due to electrostatic interactions, resulting in a thicker and more complex protein corona. This enhanced protein corona can improve the NPs’ ability to bind to negatively charged cancer cells, as it may reveal positively charged regions that facilitate strong electrostatic interactions. Conversely, negatively charged NPs form a distinct protein corona composition, often resulting in less effective binding to cancer cells. The protein corona on negatively charged NPs tends to be less complex and may not expose regions capable of strong interactions with the negatively charged surfaces of cancer cells. Understanding these dynamics is essential for the design of NPs intended for cancer therapy and diagnostics. Researchers can optimize NP interactions with cancer cells by manipulating surface charge and protein corona composition, thereby enhancing the targeting efficiency and therapeutic outcomes. This knowledge is instrumental in developing more effective NP-based systems for the sensitive detection and treatment of cancer cells in clinical settings [79,80,81,82].

Singh et al. [65] proposed a ML model to explore the influence of NM properties on cellular interactions and presented cell and nuclear shapes (CSI and NAF) as nanotoxicity markers. The study revealed that variations in epithelial cell shape are influenced by NM physicochemical properties, such as size, shape, concentration, diffusivity, zeta potential, and polydispersity, which affect intracellular uptake. They used optical methods to create nanodescriptors for analyzing cell–NM interactions, successfully predicting phenotypic markers across five NM classes. Research has highlighted how factors such as crystallinity, density, and electrical properties affect interactions and demonstrated that shape anisotropy impacts the CSI and NAF when spherical gold NPs are used as models. Ultimately, these findings suggest that NM-induced shape changes in cells may lead to epigenetic modifications and affect proliferation, emphasizing the need to consider NM properties in toxicity assessments. Fu et al. [66] highlighted the use of ML to predict the RPA of multiple proteins in the protein corona, which is important for biomedicine. Their study employed various algorithms for predicting protein adsorption to NPs and RPA values through classification and regression tasks. They utilized SHAP analysis to identify performance differences among models and reported that features such as “NP without modification” and “Incubation protein source” significantly affect RPA prediction, providing insights for protein corona design. However, they noted the challenge of modeling individual proteins separately and suggested that future research could integrate protein features for better model generalization. Overall, these findings emphasize the potential of ML in predicting the RPA of multiple proteins on the protein corona, providing valuable insights into the development of NPs for biomedicine.

William et al. [67] studied how AI can analyze protein interactions with ENMs to address potential toxicity risks. They used an AI model called the polypeptide chemical reaction optimized resistant logistic regression model (PCRO-RLRM), which enhanced protein composition predictions through Z score normalization and the position-specific scoring matrix (PSSM). The model achieved an accuracy of 96.57%, with sensitivity of 94.5% and specificity of 98.03%. These findings highlight the need for further research on protein interactions with ENMs and suggest that future studies should explore real-time interactions and integrate multiomics for deeper insights. Boadu et al. [10] reviewed DL methods for protein function prediction, highlighting advancements and challenges. They categorized these methods into four types: sequence-based, structure-based, interaction-based, and integrative methods, noting that structure and interaction methods often incorporate sequence data. The authors discussed the data sources, evaluation metrics, and critical assessment of protein function annotation (CAFA) benchmarks used to aid method development. They emphasized few-shot learning for predicting rarely annotated protein functions and outlined 30 DL methods. Additionally, they suggested developing LLMPs to address ongoing challenges. This review emphasizes the promise of DL and AI in advancing protein function prediction while calling for continued research.

### 2.2. Nanobio Interactions

Bai et al. [69] emphasized the importance of systematically exploring nanobio interactions, as many studies alter NP properties in non-systematic ways, limiting the understanding of their biological effects. Data-driven methods, such as ML, could aid in predicting these interactions and reducing the need for animal testing. Effective experimental design, along with automated synthesis and characterization, is essential for developing trustworthy models. Most investigations focus on individual property variations, neglecting the combined effects of multiple NP properties, which are often critical. Variations in preparation methods and experimental conditions further complicate comparisons across studies. Thus, more systematic research is necessary to deepen our knowledge of and accelerate the development of reliable predictive models. The complexity of nanobio interactions exceeds that of small-molecule interactions, and despite the need for faster synthesis and testing nearly a decade ago, progress has been slow, with traditional methods such as protein NMR not applicable to NPs. Yan et al. [59] utilized convolutional neural networks to predict nanobio interactions from nanostructure images, creating a novel annotation method inspired by facial recognition technology. This approach eliminates complex nanodescriptor calculations and allows accurate predictions of the physicochemical properties (logP, zeta potential) and biological activities (cellular uptake, protein adsorption) of 147 unique NPs, including platinum, palladium, and gold. The models demonstrated high accuracy, with R^2^ values exceeding 0.68 for both external and cross-validation predictions, and provided explainability through class activation maps, enhancing DL efficiency for NM design.

Hirano and Kameda [68] studied the aromaphilicity indices of amino acids and their interactions with carbon NMs, which are essential for understanding protein adsorption and bioavailability in biological systems. They introduced the “aromaphilicity index”, which measures the affinity of 20 proteinogenic amino acids for aromatic carbon surfaces, and validated it via molecular dynamics (MD) simulations. This index strongly correlated with the experimental data and effectively predicted protein binding affinities for aromatic NMs, such as CNTs and graphene. The binding free energies for amino acids were calculated via an uncapped model, which differs from previous capped approaches. The aromaphilicity index, which shows unique trends but strong correlations with the experimental results, aids in predicting protein stability and positions on aromatic surfaces, which govern the formation of the protein adsorption layer, encompassing the development of the protein corona. This tool enhances the understanding of protein interactions and potential NM applications. Jia et al. [27] emphasized that ML greatly improves the design and discovery of NMs, streamlining research compared with traditional methods that often involve costly experiments or complex calculations. ML reduces labor and time in material testing and enables high-throughput screening, making it a valuable tool for NM research. However, challenges persist in translating NMs from the laboratory to industry, including knowledge gaps and the need for better interpretability of ML algorithms. The review addresses key aspects, such as structure design, properties, and interactions with biological systems, while noting issues such as inadequate databases and algorithm accuracy that require attention for future NM development.

Rao et al. [62] examined nanotheranostic design via ML to address the limitations of traditional diagnostics and therapies. Despite advancements, challenges such as lengthy NP synthesis, incomplete understanding of nanobio interactions, and regulatory hurdles persist. ML enhances NP synthesis, uncovers new materials, and offers insights into nanobio interactions. Deep neural networks (DNNs) aid in the development of diagnostic tools, improve detection accuracy, and optimize drug delivery. While ML shows promise, further work is needed to navigate nanotechnology complexities. Collaboration across scientific disciplines is essential to leverage ML for improved clinical outcomes. Panigrahi et al. [37] examined the role of nanoinformatics in understanding the interactions between blood plasma proteins and carbon-based NMs for biomedical applications. Despite the significant growth of carbon-based NMs in biomedicine over the last thirty years, challenges remain in translating laboratory findings to clinical use owing to a limited understanding of the bio–nano interface. Key challenges include the complex interactions of NM properties (shape, size, surface chemistry) with biomolecules. The development of NMs that effectively target biological sites while minimizing interactions with proteins and lipids is difficult. However, computational methods and AI techniques, such as predictive modeling and DL, show promise for enhancing drug retention and biocompatibility. Recent studies have emphasized the importance of computational simulations in understanding these interactions, positioning nanoinformatics as a vital field in nanobiotechnology for risk assessment and exploring the theranostic potential of NMs.

### 2.3. Nanomedicines and Drug Discovery

**Figure 2:**
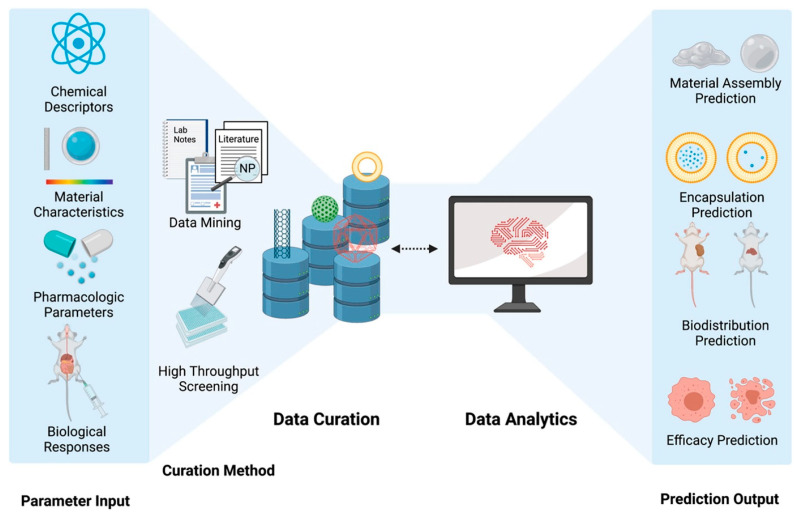
Curation workflow for nanomedicine data and data analysis [34]. Reused with permission [34]. Copyright (2022), *Elsevier*.

**Figure 3:**
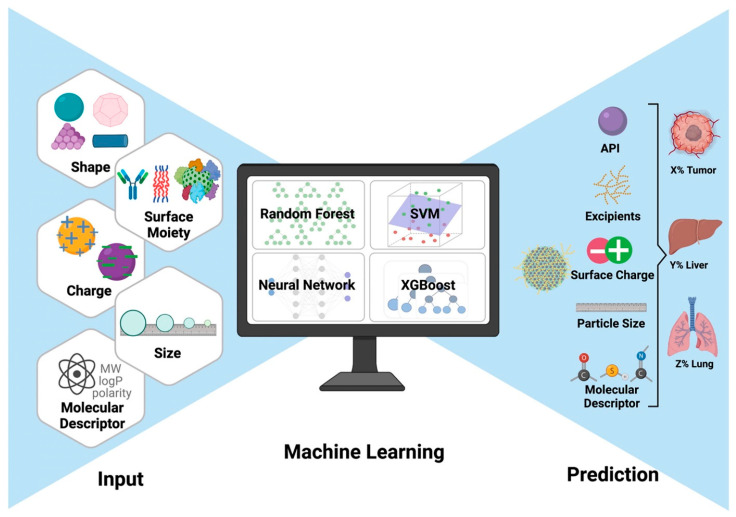
Enhancing nanomedicine efficacy: Utilizing machine-learning platforms for predictive biodistribution [34]. Reused with permission [34]. Copyright (2022), *Elsevier*.

Chen et al. [34] reviewed how integrating data curation with ML can enhance nanomedicine development, which has relied heavily on trial and error. As data science has increased in importance, recent efforts have focused on predicting the synthesis and biological behaviors of NMs through advanced analytics. ML algorithms can analyze large datasets to forecast material properties and efficacy in nanomedicine. However, collaboration between data curation and analytics is often lacking, with both fields progressing independently. This review stresses the need for cooperation between AI developers and nanoinformaticians to increase the clinical applicability of nanomedicine. This study also highlights the potential of ML for creating innovative nanomedicines and characterizing their biological interactions while addressing a significant challenge: the limited availability of diverse data for effective algorithm training. Figure 2 illustrates the curation workflow for nanomedicine data and data analysis. Figure 3 shows the enhancement of nanomedicine efficacy via the use of ML platforms for predictive biodistribution.

**Figure 4:**
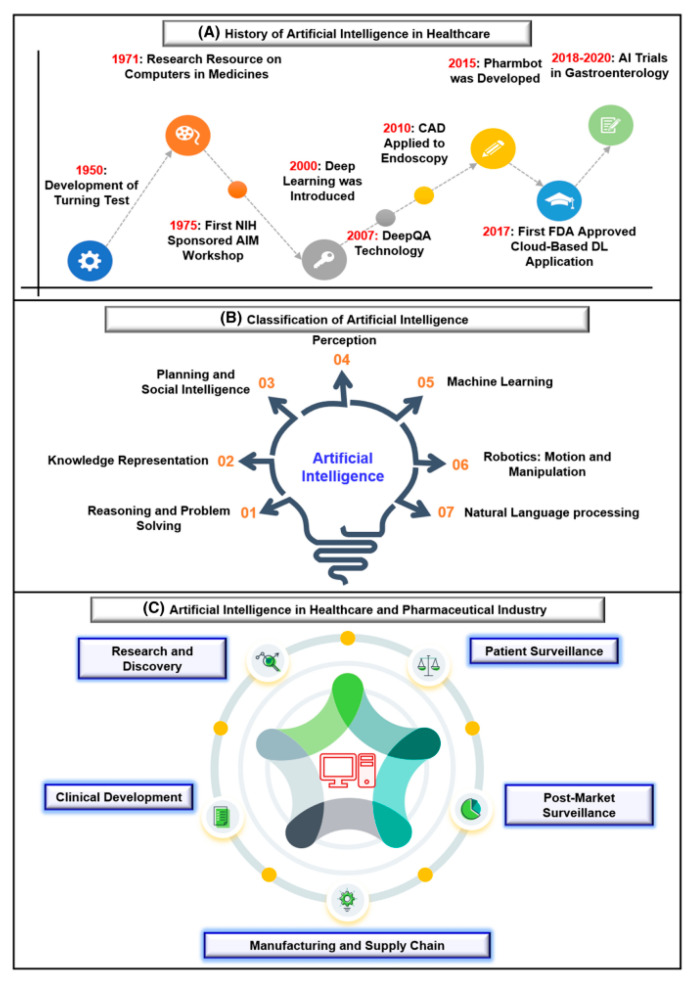
Advancements and applications of artificial intelligence in healthcare and pharmaceuticals [72]. Reused with permission [72]. Copyright (2021), *Springer Nature*.

Gupta et al. [72] explored the role of AI and DL in drug discovery, highlighting the transition from ML to DL and the impact of big data. They reviewed how AI integrates with traditional chemistry to enhance various stages of drug development, including screening, toxicity assessment, drug dosage effectiveness and efficacy, drug release and monitoring, drug repositioning, drug–target interactions, and polypharmacology. Overall, advancements in AI and DL offer significant opportunities for improving rational drug design and discovery, benefiting humanity. The advancements and applications of AI in healthcare and pharmaceuticals are prominently illustrated in Figure 4.

Dhakal et al. [6] reviewed the role of AI in predicting protein–ligand interactions, highlighting its importance in drug discovery. They provided an overview of proteins, ligands, and relevant databases and analyzed various ML approaches for predicting protein–ligand binding sites, ligand-binding affinities, and binding poses. The authors emphasized the potential for improved prediction accuracy by integrating diverse physicochemical properties and DL techniques, suggesting a multitask approach to unify these traditional separate tasks to increase drug discovery efforts. Scott-Fordsmand and Amorim [71] reviewed the use of ML to increase the sustainability of NMs in environmental risk assessment (ERA). They noted that while ML can improve data gathering, exposure assessment, hazard identification, and risk characterization, its application and standards are insufficient. Emphasis is needed on data quality and addressing historical biases to prevent errors. The review also noted that the advantages of ML over traditional methods are often overstated, and comprehensive integration of ML at all ERA levels is lacking. Understanding material descriptors for smart NMs is essential, as different factors may influence various risks, such as size for cellular uptake and surface charge for environmental fate.

Wang et al. [70] reviewed the barriers to the systemic delivery of NPs, highlighting the importance of effective in vivo delivery for successful nanomedicine. They introduced the concept of “NP blood removal pathways” (NBRP), which encompass various cell-dependent and cell-independent blood clearance mechanisms. This review emphasizes NP design and biological modulation strategies to increase delivery by reducing NBRP interactions, noting that surface chemistry is a critical factor. Combinatory biological-PEG surface modifications increased blood circulation by approximately 418% and decreased liver accumulation by up to 47%. Strategies to overcome biological barriers are categorized into those that modify NP characteristics and those that alter the biological environment. A better understanding of NP–NBRP interactions can lead to safer and more efficient nanomedicines. The review also identifies opportunities for improvement, such as standardizing design and reporting, utilizing central data repositories, and applying meta-analysis and ML in future nanomedicine development. Nguyen et al. [73] reviewed how AI enhances G-protein-coupled receptor (GPCR) drug discovery, supporting stages from understanding GPCR functions to identifying ligand interactions and predicting clinical responses. They covered key AI concepts, including ML and DP, existing applications, and discussed the benefits and limitations of AI in GPCR drug discovery, expressing optimism about its potential to make drug discovery faster, smarter, and more cost-effective.

### 2.4. Protein–Protein Interactions

The dynamics of PPIs are influenced primarily by the structural characteristics of their binding interfaces [83]. Research has shown that mutations in residues located at the interface of protein complexes can considerably alter their stability, thereby impacting their cellular functions [84]. Consequently, elucidating the structural basis of protein interactions is essential for gaining a deeper understanding of the functions of these molecules [19]. Despite advances in experimental data and computational resources, our understanding of PPI is still limited. The complexity of PPIs, which are influenced by transient aggregates and non-transients, different compartments, and macromolecular condensates, complicates the interpretation of proteomic data. The current models, even those at atomic resolution, often capture only a small fraction of functional interactions in crowded cellular environments. Distinguishing functional from non-functional interaction surfaces remains difficult. While ML and DL show promise in many areas, their application in PPI predictions has limitations, with both shallow and DL methods yielding similar results, indicating insufficient representations of interacting surfaces and varying binding affinities on the basis of cell type and regulation. More high-resolution examples of protein complexes are needed to address these challenges [2].

**Figure 5:**
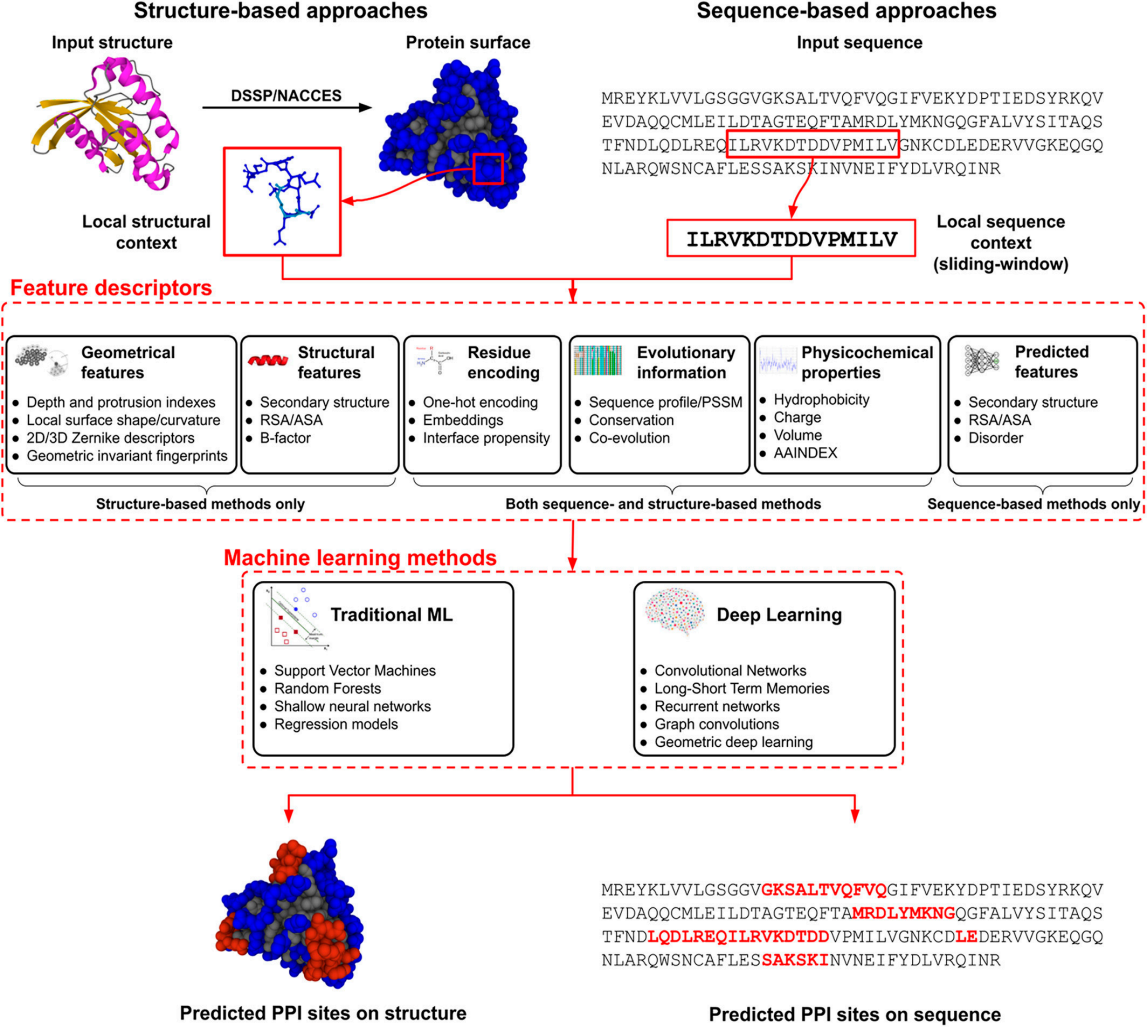
Schematic representation of ML techniques for predicting PPI sites based on structure and sequence. [2]. Reused from reference [2]. Copyright R. Casadio, P.L. Martelli, C. Savojardo, 2022 [2]. Some rights reserved; exclusive licensee [John Wiley and Sons]. Distributed under a Creative Commons Attribution License 4.0 (CC BY) https://creativecommons.org/licenses/by/4.0/ (accessed on 8 March 2025).

**Figure 6:**
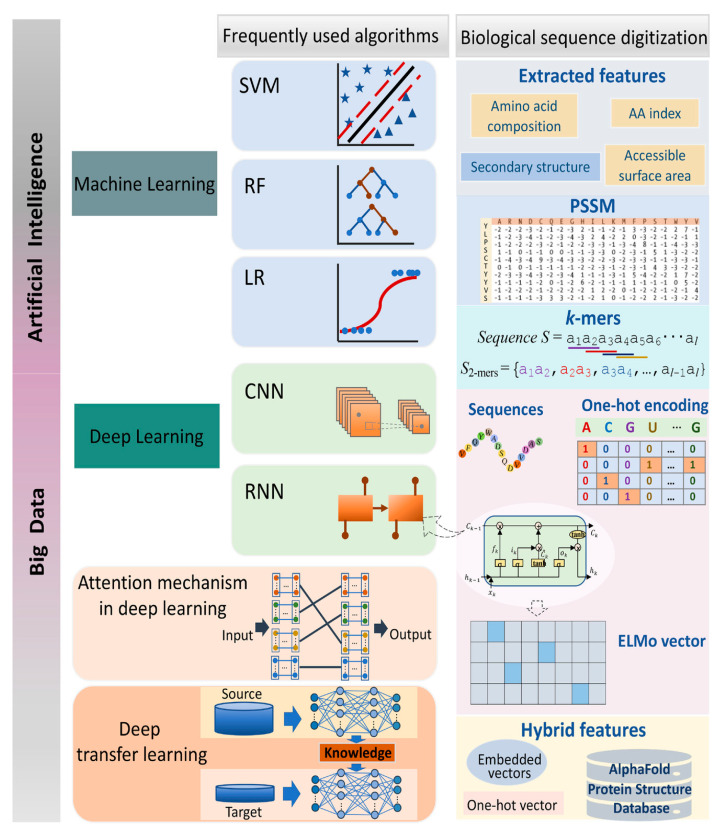
Advancements in machine-learning algorithms for analyzing DNA/RNA–protein interactions [64]. Reused with permission [64]. Copyright (2022), John Wiley and Son.

Jovine [78] explored ML applications in analyzing PPIs, particularly UMOD polymerization. Using advanced neural network models, such as AlphaFold2 and ColabFold, this study achieved near-experimental accuracy in protein structure prediction. It successfully predicted a crucial conformational change in UMOD without training on the polymer structure. By simulating propeptide dissociation due to proteolysis, this research highlights the potential of ML in clarifying complex molecular events and provides insights into egg coat protein assembly and ZP module-containing molecules. Casadio et al. [2] reviewed ML solutions for predicting PPIs, highlighting the role of proteins as “social molecules”. Recent studies have shown that large protein aggregates, or biomolecular condensates, play significant roles in various biological processes. These condensates can be either permanent or time-dependent and are influenced by cellular needs. However, monitoring protein aggregate formation poses challenges, both experimentally and theoretically, especially in predicting functional aggregates. The current research includes mesoscopic networks at the proteome level, protein-binding affinities, and atomic-resolution complexes. While ML algorithms can derive insights from data, they need rigorous benchmarking on blind datasets for validation. Even advanced ML methods, such as DP, require further training on the full range of PPIs. Although PPIs are crucial for processes such as transcription and protein biosynthesis, the transient complexes that form condensates are less understood. A key question is how to differentiate functional PPIs from non-specific aggregates. Our understanding relies on atomic-level complex data from the Protein Data Bank (PDB) and broader analyses of protein complex formation. The role of ML in PPI challenges, available data resources, and predicting PPI networks, 3D aggregates, and PPI sites on structures and sequences are also discussed. Figure 5 provides a schematic representation of the ML techniques employed for predicting PPI sites, utilizing both structural and sequence-based information. Cui et al. [64] reviewed protein–DNA/RNA interactions, focusing on the evolution of computational methods in proteomics from traditional ML to DL, driven by advancements in AI and big data. This review discusses the tools for predicting interactions, their advantages, shortcomings, and applications, along with biological sequence-digitizing strategies and data representation challenges. The authors suggest that future research could integrate various AI approaches, including DL, reinforcement learning, and evolutionary methods. Figure 6 illustrates the advancements in ML algorithms used for analyzing interactions between DNA/RNA and proteins.

Hong et al. [74] introduced PhosPPI, a sequence-based ML approach for predicting the impact of phosphorylation on PPIs. Phosphorylation is vital for cell signaling and can contribute to diseases such as cancer and Alzheimer’s disease. PhosPPI addresses the challenge of experimentally determining these effects and outperforms existing methods, such as Betts, HawkDock, and FoldX, in accuracy. It functions without the need for protein 3D structures, making it more accessible. With strong validation performance, PhosPPI is a valuable tool for biologists and bioinformaticians in studying the role of phosphorylation in PPIs and drug development.

Lee [77] reviews recent advances in DL for PPI analysis, highlighting its transformative role in computational biology. The review covers the literature from the period 2021–2023 and highlights innovative methodologies that are essential for understanding biological systems and therapeutic opportunities. These findings underscore the need for ongoing adaptation in DL applications, providing key insights into how these techniques are transforming PPI predictions and advancing biological research and therapeutic strategies. Ye et al. [75] reviewed advances in ML for predicting peptide/protein–protein interactions (PepPIs/PPIs) via sequence data, emphasizing their role in drug discovery. This paper discusses high-throughput technologies, ML methods for lead peptide discovery, and various databases of peptide ligands and target proteins. It categorizes classical ML and DL approaches, evaluates their advantages and disadvantages, and covers validation protocols and performance metrics. This review highlights the importance of PPIs in biological processes and the need for computational models to enhance predictions, given the limitations of laboratory methods. This study concludes with insights into challenges and future directions to improve bioactive peptide and protein discovery for drug development.

Sousa et al. [76] studied PPIs via a knowledge-graph-based method called KGsim2vec, which generates explainable vector representations by leveraging aspect-oriented semantic similarity. While AI and ML are increasingly applied in biomedical fields, ensuring explainability is essential for scientific discovery. Their approach improves upon typical knowledge graph embeddings by enhancing explainability and predictive performance with ML models, such as decision trees and random forests. They also addressed challenges in knowledge graph representations and introduced an innovative method for evaluating explanation quality, achieving significant results in the prediction of PPIs. Su et al. [19] investigated PP binding interfaces via AI, revealing high similarity among these interfaces in various complexes. They decomposed binding interfaces into interacting fragment pairs and applied a generative model to encode them in a low-dimensional latent space. After training, they generated new fragment conformations, aiding in the assembly of native protein complexes. These findings indicated that the conformational space of these pairs is highly degenerate and can be effectively characterized by AI. By developing a generative autoencoder and clustering samples with a self-organizing map (SOM), they reported that most generated pairs resembled native-like structures, suggesting the potential for predicting unknown PPIs.

## 3. Machine-Learning Training Models and Databases in the Field of Protein Interactions, Drug Discovery, and Bioinformatics

A variety of ML models are employed to predict interactions in GPCR drug discovery, including random forests (RFs), neural networks (NNs), support vector machines (SVMs), and extreme gradient boosting (XGBoost). Table 4 compares these models, highlighting their respective advantages and limitations. Each model has distinct strengths and weaknesses, making them suitable for different problems and datasets. The selection of an appropriate model is contingent upon the specific requirements of the task, such as the size and nature of the dataset, the complexity of the relationships within the data, the need for interpretability, and the computational resources at hand [2,6,10,64,66,67,73,75,77]. On the other hand, hybrid models in ML integrate various algorithms to leverage their unique strengths while minimizing their individual weaknesses, leading to enhanced prediction accuracy and robustness. For instance, combining RF with NN creates a powerful framework that excels in complex prediction tasks. This hybrid approach benefits from the interpretability and stability of RFs alongside the advanced pattern recognition capabilities of NNs. In a typical hybrid model, RFs can be used for feature selection and preprocessing, effectively identifying the most relevant inputs. NNs, on the other hand, excel at extracting high-level features from raw data, allowing the model to learn intricate patterns that might otherwise be overlooked. This two-step process not only takes advantage of deep feature extraction but also enhances the decision making of RFs, leading to improved generalization of unseen data. Moreover, combining different models helps mitigate overfitting, as the ensemble nature of RFs can smooth out the predictions made by NNs, which might overfit training data. Hybrid approaches can significantly reduce the overall error rate by addressing the different error types often produced by individual models. For example, if an NN makes systematic errors in certain areas, an RF may correct those errors, ensuring a more robust prediction. These hybrid strategies are particularly valuable in domains requiring high precision and reliability, such as drug discovery and bioinformatics. By effectively managing complex datasets and minimizing prediction errors, hybrid models provide a solid solution for tackling challenging tasks, resulting in more accurate and trustworthy predictions [2,6,10,64,66,67,73,75,77]. 

Numerous databases are available for research in AI and ML applications specifically focused on PPIs, peptide–protein interactions (PepPI), and GPCR drug discovery. Some key databases and their applications are shown in Table 5. Understanding the available databases and their applications is essential for researchers engaged in these fields. Utilizing these databases is crucial for acquiring high-quality data, extracting relevant features, and validating predictive models. These databases serve as invaluable resources, supplying the data needed to train and validate ML models and enabling the extraction of pertinent features. They support various aspects of drug discovery, including virtual screening, de novo drug design, and drug repurposing. A thorough understanding of these databases can significantly enhance the efficiency and accuracy of research efforts in this domain. By comprehending the applications of these databases, researchers can develop more precise and robust ML models, thereby advancing our understanding of PPIs. These resources are fundamental for training, validating, and benchmarking ML models, ultimately facilitating the discovery of new interactions and therapeutic targets [2,6,10,62,73,75,77]. 

## 4. Opportunities, Limitations, and Challenges in the Application of AI and ML Techniques for Characterizing Protein Corona, Nanobio Interactions, Nanomedicines and Drug Discovery, and Protein–Protein Interactions

The opportunities, limitations, and challenges are outlined and discussed in Table 6. They are categorized under the following headings: nanotoxicology and nanomaterial research, protein corona prediction, nanomedicine and drug discovery, protein function prediction, nanobio interactions and nanoinformatics, environmental risk assessment, drug discovery and development, PPIs, explainable AI, and DL for PPI analysis.

The document outlines opportunities for advancing protein function prediction via ML and AI. This highlights the importance of understanding protein–NM interactions, with a focus on protein corona prediction and applications in nanomedicine. The key areas for enhancement include refining ML algorithms for accurate predictions of nanomaterial–cell interactions, utilizing interpretable ML for decision-making processes in protein corona prediction, and developing safer nanoparticles for biosensing and organ targeting. Additionally, it suggests the need to create large LLMPs, leverage high-throughput methods to generate extensive datasets for ML model development, and incorporate AI in exposure assessment and risk characterization. The document also emphasizes the need to integrate omics data to understand disease mechanisms, improve drug discovery processes, and enhance the methods for studying PPIs while ensuring explainability and scalability in AI models. By addressing these opportunities, researchers can advance the understanding and application of nanotechnology, protein interactions, and AI in various fields. Advanced computational tools and physicochemical analyses can enhance the safety assessment of NMs. ML can significantly improve protein function prediction and the understanding of protein coronas. The aromaphilicity index offers a tool for predicting protein–NM interactions. Additionally, ML can revolutionize nanotheranostics, leading to better patient outcomes through improved diagnostic and therapeutic strategies. Overall, these advancements aim to enhance nanobio interactions, improving the efficacy and clinical translation of nanomedicines across biomedicine, environmental science, and consumer products.

The document highlights several challenges and limitations in nanotoxicology and NM research, protein corona prediction, nanomedicine and drug discovery. In nanotoxicology, the complex interactions between NMs and biological systems hinder accurate predictions of behavior and toxicity, compounded by inherent heterogeneity, variability in biological responses, and a lack of understanding of chronic exposure effects. For protein corona prediction, modeling individual proteins limits generalization, whereas complex neural networks may overfit and lack feature fusion during training. In nanomedicine and drug discovery, the challenges include the need for large, high-quality datasets for training ML models, inconsistent reporting metrics, and time-consuming manual data curation. Overfitting of ML models is common due to the complexity of nanomaterials. For protein function prediction, difficulties arise from integrating diverse data modalities, utilizing evolutionary information, and maintaining model scalability, alongside the challenge of curating high-quality datasets. For nanobio interactions and nanoinformatics, comprehending the intricate interactions between NPs and biological systems is hindered by non-systematic studies and a lack of reliable datasets. The transformation of NPs in biological environments adds further complexity. In environmental risk assessment, high-quality data are often incomplete, and ML models can reflect biases and errors from their training data. Their complexity and “black box” nature challenge interpretability and reproducibility, which affects drug discovery and regulatory approval. For PPIs, accurately representing complex protein sequences is challenging. ML models may generate false positives, but the dynamic nature of PPIs complicates analysis. Explainable AI faces obstacles due to the complexity of data in the biomedical domain, and knowledge graphs often lack specific interpretations. While promising, DL models struggle with data harmonization and structural predictions of protein complexes. Addressing these challenges can increase the accuracy and applicability of ML and AI in scientific and medical fields.

## 5. Conclusions

In this review, recent advancements in ML and DL methodologies employed to elucidate protein corona characterization, nanobio interactions, nanomedicine development, drug discovery processes, and PPIs are reviewed. The advantages and limitations inherent in these approaches, their diverse applications across various fields, and prospective future trends that may emerge in this rapidly evolving domain are critically examined. The documents highlight advancements in the use of AI to model and predict PPIs, presenting a promising tool for the field of computational biology. The results underscore the importance of ML in understanding how proteins assemble and suggest potential future applications in the exploration of other complex biological systems. Additionally, they emphasize the transformative impact and potential of AI and ML in drug discovery and development while also acknowledging ongoing challenges and the need for continued progress in this area. These findings provide a comprehensive overview of the challenges and strategies for improving NP delivery in nanomedicine, highlighting the importance of both NP design and biological modulation. This information serves as a valuable resource for researchers, helping them to navigate the latest advancements and methodologies in this rapidly evolving field.

Several key conclusions across various fields involving ML and AI are as follows:

- *Nanotoxicology and nanomaterial research*: ML algorithms can effectively predict cell and nuclear shapes and polarity functions as phenotypic markers for diverse categories of NMs, aiding in understanding NM interactions with biological systems. Establishing standardized protocols and interdisciplinary collaboration is crucial for advancing nanotoxicology research.
- *Protein corona prediction*: ML models can predict the RPA of multiple proteins on the protein corona, reducing the need for traditional experimental techniques. The key attributes associated with RPA analysis have been identified, providing insights for protein corona design. Future improvements should focus on integrating more comprehensive datasets and advanced ML techniques.
- *Nanomedicine and drug discovery*: ML and AI significantly enhance the design and application of NPs in nanomedicine, biosensing, and organ targeting. Combining data from various omics fields can provide a comprehensive understanding of NM interactions. Real-world validation of ML models is essential to ensure their accuracy and reliability in practical applications.
- *Protein function prediction*: DL methods have improved protein function prediction, but challenges remain in integrating multiple data modalities and predicting rare or novel functions. Collaboration among the ML, AI, and bioinformatics fields is necessary to develop cutting-edge techniques for predicting protein functions.
- *Nanobio interactions and nanoinformatics*: Systematic exploration of the physicochemical properties of NPs and their interactions with biological systems is needed. High-throughput synthesis and testing methods, combined with advanced computational models, can accelerate the comprehension of nanobio interactions. Effective data management and sharing protocols are essential for handling large datasets.
- *Environmental risk assessment*: ML can enhance ERA by improving data gathering, exposure assessment, hazard identification, and risk characterization. The development of comprehensive strategies and standard controls for ML in the ERA is crucial for ensuring accuracy and reliability.
- *Drug discovery and development*: AI has the potential to greatly reduce both the time and expenses associated with drug discovery by automating and optimizing various stages of the process. AI-driven predictive modeling and simulation can enable virtual testing of drug candidates, accelerating the drug development process. Ethical and regulatory frameworks are needed to promote the ethical application of AI in drug discovery.
- *Protein–protein interactions*: ML models can predict PPIs with increased accuracy by integrating multiple data sources and leveraging advanced computational techniques. Moving beyond interaction prediction to understand the functional and contextual relevance of PPIs is a significant advancement. Combining sequence features, docking scoring functions, and protein binding site predictions can increase the accuracy of AI models.
- *Explainable AI*: Explainability is crucial for the adoption of ML as a scientific tool, ensuring reliability and interpretability. Ontologies and knowledge graphs can improve the interpretability of ML applications in the biomedical domain. The development of scalable methods to handle large datasets and complex knowledge graphs efficiently is essential.
- *Deep learning for PPI analysis*: DL presents a transformative platform for predicting PPIs, significantly advancing our understanding of protein interactions and biological systems. Addressing challenges such as data quality, model interpretability, and the integration of diverse data sources is crucial for further advancements. The application of DL models to interdisciplinary domains, such as drug discovery and personalized medicine, holds significant potential.

Overall, the document emphasizes the transformative potential of ML and AI in various scientific and medical fields while also acknowledging the ongoing challenges and the need for continued progress and collaboration.

## 6. Future Perspectives

The document presents various future directions for advancing the field of nanotoxicology nanomaterial research, protein function prediction, nanomedicine, drug discovery, and PPIs via ML and AI:

- *Nanotoxicology and nanomaterial research*: Enhance ML algorithms for better prediction of NM–cell interactions; include diverse cell types and models to study NM effects; standardize the protocols for reporting NM interactions with biological systems; address the need for standardized nanodescriptors in ML models to accurately predict the behavior of NPs; and foster collaboration among material scientists, biologists, toxicologists, and computational experts.
- *Protein corona prediction*: Enhance feature representation by extracting additional protein-related features; create generalized models using comprehensive datasets and advanced ML techniques; investigate advanced neural network architectures to prevent overfitting and increase performance; build integrated models for efficient classification and regression; and prioritize interpretable ML methods for better decision-making transparency.
- *Nanomedicine and drug discovery*: Combine data from genomics, proteomics, and metabolomics for a comprehensive understanding of NM interactions; optimize computational models such as PCRO-RLRM for enhanced predictive performance; develop advanced DL and AI techniques to integrate diverse input data; use evolutionary data from protein sequences to enhance function prediction; create next-generation LLMPs tailored for multimodal data and function prediction; and foster collaboration among ML, AI, and bioinformatics for innovative protein function prediction methods.
- *Nanobio interactions and nanoinformatics*: Explore the physicochemical properties of NPs and their biological interactions; use combinatorial chemistry and high-throughput methods to create diverse datasets; develop computational models to predict NP biological effects; accelerate data generation on nanobio interactions through high-throughput synthesis and testing; and enhance data management and sharing for the effective handling of large datasets.
- *Environmental risk assessment*: Develop strategies to implement ML in ERA, focusing on data foundations and methodologies; standardize and validate ML models for accuracy and reliability; integrate ML with the IoT for real-time environmental monitoring; and target complex data areas, such as spatial–temporal analysis and omics.
- *Drug discovery and development*: AI will increase the efficiency and accuracy of drug design, leading to the discovery of new therapeutic compounds; it will aid in customized healthcare through the examination of individual patient data for tailored treatments; AI will integrate omics data for a thorough comprehension of disease mechanisms and drug responses; AI-driven predictive modeling and simulations will allow virtual testing of drug candidates, reducing the need for extensive experiments; clinical trials will be optimized through better patient selection, outcome prediction, and real-time monitoring; AI will facilitate drug repurposing by finding new uses for existing medications; quantum computing will significantly advance AI’s role in drug discovery; and it is essential to develop ethical and regulatory frameworks for responsible AI use in this field.
- *Protein–protein interactions*: Develop ML algorithms, including DL, to improve PPI prediction accuracy; integrate atomic resolution structures with proteome-wide interaction networks for comprehensive predictions; increase the representation of protein sequences and structures for ML models; improve the computational efficiency of ML models for broader access to advanced techniques; and focus on the functional and contextual relevance of PPIs in various cellular environments.
- *Explainable AI*: Developing scalable methods for large datasets and complex knowledge graphs; improving feature extraction and selection from diverse data sources; enhancing model transparency via attention mechanisms and saliency maps; and applying DL models in drug discovery, personalized medicine, and environmental genomics.
- *Deep learning for PPI analysis*: Refine fold-and-dock algorithms for better protein pair structure predictions; integrate sequence features, docking scores, and protein binding site predictions into AI models; use advanced computing resources for training AI on larger datasets; and apply AI models in drug discovery to identify targets and design therapies.
- *General future directions*: Create centralized databases for data sharing and collaboration; implement real-time monitoring of nanomedicine performance; focus on developing safe, sustainable nanomaterials; conduct in vivo studies to understand protein corona dynamics; use AI and ML to analyze nanobio interaction data; and develop in silico models to predict nanobio interactions.

Addressing future perspectives in ML and AI can significantly enhance their application in scientific and medical fields, particularly in drug discovery and development. Continued progress in AI, along with ethical considerations and collaboration, promises to transform the pharmaceutical industry and improve global health outcomes. The key future directions for ML include strategies for standardizing models, addressing data biases, integrating with the IoT, and fostering interdisciplinary collaboration. These efforts will advance nanomedicine and lead to more personalized treatments. There is also great potential for innovations in nanotheranostics, improving disease diagnosis, imaging, and therapy. Additionally, focusing on the aromaphilicity index and protein corona prediction can enhance our understanding of nanotechnology in the biological and medical sciences. In nanotoxicology, these advancements aim for safer NM applications. Overall, prioritizing multimodal integration and community collaboration is crucial for enhancing protein function prediction and advancing the field of nanomedicine.

## Figures and Tables

**Table 1:** Key factors influencing protein corona formation.

Key Factors	
*Nanoparticle characteristics*	The critical factors influencing NPs include their size, surface chemistry, charge, and shape.
*Physicochemical properties*	The physicochemical properties of NPs include their hydrophilic or hydrophobic characteristics, solubility, and surface functionality.
*Environmental conditions*	The biological environment is influenced by factors such as temperature, pH, and ionic strength.
*Protein binding affinities*	Different proteins have varying affinities for NPs, influencing the composition and stability of the protein corona.
*Exposure time*	The duration of NP exposure to the biological environment affects the dynamic nature of the protein corona.
*Vroman effect*	Initially adsorbed proteins with lower affinity are replaced by higher affinity proteins over time.
*Protein concentration*	The abundance of proteins in the biological medium can impact the formation and composition of the protein corona.

**Table 2:** Impact of protein adsorption on nanoparticle surface interactions.

Factors	Influence
***Surface properties***	Protein adsorption can change the surface chemistry, charge, and hydrophobicity/hydrophilicity of nanoparticles, affecting their interaction with biological systems.
***Stability***	Protein adsorption can either stabilize or destabilize NPs. It can prevent aggregation by providing a steric barrier or induce aggregation if the proteins cause cross-linking between particles.
***Biocompatibility***	The type and amount of proteins adsorbed can influence the biocompatibility of nanoparticles, potentially reducing or increasing their toxicity and immunogenicity.
***Cellular uptake***	The protein corona formed by adsorbed proteins can affect how NPs are recognized and internalized by cells, impacting their efficiency in drug delivery and other therapeutic applications.
***Circulation time***	Adsorbed proteins can influence the circulation time of NPs in the bloodstream, affecting their ability to reach target sites before being cleared by the body.
***Targeting and functionality***	Protein adsorption can mask or alter the functional groups on the NP surface, potentially interfering with their ability to bind to specific target cells or tissues and perform their intended function.
***Toxicity***	The adsorption of certain proteins can mitigate or exacerbate the toxic effects of NPs, influencing their safety profile in biomedical applications.

**Table 3:** Overview and main findings of the literature evaluations on the application of AI and ML techniques for characterizing protein corona, nanobio interactions, nanomedicines and drug discovery, and protein–protein interactions.

Overview	Main Findings	References
Protein Corona Characterization		
The study examines a ML method to decode the relationship between NM properties and cell interactions, focusing on cell shape index and nuclear area.	The physicochemical properties of NMs, including shape, size, and surface charge, significantly impact cell and nuclear shape indices (CSI and NAF). A ML approach effectively predicted cell and nuclear shapes as phenotypic markers for various NM classes. Different NMs cause distinct changes in epithelial cell shape and nuclear positioning, linked to their properties. CSI and NAF can serve as intuitive phenotypic parameters to evaluate NM safety in consumer products and nanomedicine. The actin cytoskeleton is crucial for mechanotransduction, affecting nuclear and cell shape stability in response to NM exposure. Changes in cell and nuclear shapes due to NMs impact essential cellular functions like proliferation, particularly with carbon, dendrimer, and GNRs. NM exposure alters chromatin organization, indicating possible genotoxicity through nuclear shape changes. The study recommends integrating CSI and NAF shape coupling into minimum reporting criteria for NM biological characterization to enhance nanotoxicology protocols.	Singh et al. [65]
The document is a research article investigating the use of ML to predict the RPA of proteins on the protein corona of NPs, which is vital for their biomedicine applications.	The study used six ML algorithms (ERT, RF, GBDT, XGB, LGBM, and NN) to predict RPA on protein corona. Extremely randomized trees (ERT) excelled in binary classification tasks for predicting protein adsorption to NPs, outperforming RF and GBDT across all evaluation metrics (AUROC, recall, precision, F1, MCC, and accuracy). In regression tasks, RF achieved the best R^2^ metric, while ERT excelled in RMSE. The study retained 129 baseline models for further analysis and utilized SHAP for insights into ERT and NN learning patterns, identifying “NP without modification” as a significant feature. ERT effectively transferred learning to the test set, whereas NN faced overfitting. Key features like “NP without modification” and “Incubation protein source” were identified for designing protein coronas. Oversampling enhanced ERT’s performance in some datasets. Overall, the study offers a predictive tool for RPA, potentially lowering the design cost of protein coronas.	Fu et al. [66]
The document analyzes protein compositions in ENMs using a novel AI approach called the PCRO-RLRM to predict these compositions.	The PCRO-RLRM outperformed existing methods like random forest (RF), K-nearest neighbor (KNN), and support vector machine (SVM) in predicting protein compositions on engineered nanomaterials (ENMs), achieving high accuracy, sensitivity, and specificity. Utilizing Z score normalization and PSSM for feature extraction proved effective in analyzing protein compositions. This novel approach can enhance biochemistry findings related to ENMs and improve bioengineering by deepening our understanding of protein–NM interactions, which is crucial for environmental safety, human health, and technology development.	William et al. [67]
The document reviews DL methods for protein function prediction, addressing recent advancements, challenges, and future directions. It analyzes the use of various data sources—sequences, structures, and interactions—and categorizes DL techniques into sequence-based, structure-based, interaction-based, and integrative approaches, detailing the models used in each category.	Significant progress has been made in using DL to predict protein functions from diverse data sources, including sequences, structures, and interactions. DL methods are categorized into four types: Sequence-based: Focusing on protein sequences; Structure-based: Utilizing structural information; Interaction-based: Using PPI data; Integrative: Combining multiple data sources for better predictions. Challenges remain, such as the need for effective integration of data types, difficulty in improving accuracy for rare or novel Gene Ontology (GO) terms, better exploitation of evolutionary information from protein sequences. Future directions include developing LLMPs for protein function prediction, applying few-shot and zero-shot learning techniques for rare functions, utilizing advanced AI methods from NLP to enhance predictions. The review emphasizes the use of standardized benchmarks like CAFA for evaluating prediction methods.	Boadu et al. [10]
**Nanobio Interactions**		
The document examines the aromaphilicity index of amino acids and presents MD simulations related to protein binding affinity for carbon NMs, including CNTs and graphene. An “aromaphilicity index” was developed to quantify the affinity of 20 amino acids for aromatic carbon surfaces, aiding in the prediction of protein binding hotspots on NMs, which is crucial for assessing their bioavailability and potential cytotoxicity.	The researchers developed an “aromaphilicity index” to quantify amino acid affinity for aromatic carbon surfaces like CNTs and graphene through MD simulations. Amino acids with planar side chains, such as tryptophan (Trp), tyrosine (Tyr), phenylalanine (Phe), and histidine (His), show high affinity due to van der Waals forces and π–π interactions. The binding free energy calculations revealed that aromatic amino acids and arginine (Arg) exhibit strong binding affinity. The binding affinity increases with decreasing CNT curvature, and the chiral angle has a marginal effect. The index demonstrated a strong correlation (R^2^ = 0.789) with experimental data, underscoring its practical utility. It can predict protein binding stability on aromatic surfaces, aiding in understanding protein corona formation and the effects of NMs. The index is versatile and applicable across various aromatic surfaces, with potential use in biomedicine, biosensing, and drug delivery.	Hirano and Kameda [68]
The document presents a novel approach for predicting nanobio interactions using convolutional neural networks (CNNs) to analyze nanostructure images. This method simplifies the analysis by transforming nanostructures into images, allowing for direct learning of features without complex calculations.	A new technique was developed to transform nanostructures into images for CNN modeling, inspired by face recognition technology. Features from NP images were directly learned without complex calculations. CNN models accurately predicted physicochemical properties (logP, zeta potential) and biological activities (cellular uptake, protein adsorption) for 147 unique NPs. The models achieved a determination coefficient (R^2^) over 0.68 for both cross-validation and external predictions. The model allows visualization of learning through the class activation map. This method provides an efficient pathway for designing next-generation NMs.	Yan et al. [59]
The document examines the role of ML in enhancing the design and discovery of NMs. It notes that traditional methods involve costly experiments, while ML can streamline material testing and enable high-throughput screening. The discussion includes improvements in NM structure design, properties, adsorption, and catalysis, as well as challenges related to nanobiology and the interactions of NMs with biological systems.	ML significantly speeds up material testing, allowing for high-throughput screening. ML enhances the design of NM structures, properties, adsorption, and catalysis. Analysis of ML predictions of NMs and biological system interactions reveals emerging challenges. Improving the interpretability of ML algorithms is essential, as it remains a key bottleneck. Challenges like imperfect databases, algorithm accuracy, and nanopattern recognition persist. The document highlights how ML can advance NM development while also identifying areas for improvement.	Jia et al. [27]
The document reviews the use of ML in designing nanotheranostics for improved disease management. It highlights the integration of ML with nanotechnology to enhance the development of nanotheranostics, which combines diagnostic and therapeutic functions. This approach offers benefits like improved drug delivery, reduced toxicity, and real-time treatment feedback.	ML has the potential to significantly enhance nanotheranostics by optimizing NP synthesis, decoding nanobio interactions, and predicting therapeutic outcomes. Nanotheranostics offers advantages over traditional methods, such as improved drug delivery, reduced toxicity, and real-time feedback on treatment efficacy. However, its widespread adoption faces challenges, including time-consuming NP synthesis, incomplete understanding of nanobio interactions, and difficulties in clinical translation. ML models, including NNs networks, have been effective in predicting and optimizing various types of NPs and understanding nanobiomolecule interactions. This has improved clinical detection, molecular imaging, and treatment strategies. The review emphasizes the need for large, well-annotated datasets for effective ML training and discusses challenges related to data diversity and interpretability. Future developments in ML-aided nanotheranostics are expected to enhance NP preparation, the understanding of complex nanobio interactions, and clinical outcomes.	Rao et al. [62]
The document focuses on the interaction of blood plasma proteins with carbon-based NMs (CBNs) and their implications for biomedical applications. It highlights the growing use of CBNs in drug delivery and diagnostics while addressing challenges in translating lab research to clinical settings due to the complex nanobio interface. This interface involves the formation of a biocorona that affects protein function, cellular interactions, and toxicity. Computational simulations, including MD and DFT, are emphasized as vital tools in understanding these interactions, contributing to the field of nanoinformatics. The document also covers the classification of CBNs by structure and the need for advanced techniques to study nanobio dynamics. It underscores the importance of nanoinformatics in enhancing nanobiotechnology for safe and effective biomedical applications.	There has been significant interest in CBNs for drug delivery and diagnostics over the past three decades. Translating laboratory research on CBNs to clinical applications is challenging due to complex nanobio interactions. CBNs interact with biological systems, forming a biocorona that can alter protein function, impacting cellular interactions and toxicity. Computational methods like MD and DFT are essential for understanding the nanobio interface and enhancing biomedical functionality. Nanoinformatics, a multidisciplinary field, helps analyze NM interactions with biological systems, providing insights that complement experimental methods. CBNs are categorized based on geometrical structures into 0D, 1D, 2D, and 3D forms, each with distinct properties. Advanced quantitative techniques with ultra-high resolution are needed to analyze dynamic interactions at the nanobio interface. Understanding CBN interactions with plasma proteins is crucial for assessing biocompatibility and the behavior of NPs in biological systems.	Panigrahi et al. [37]
The article reviews the systematic exploration of nanobio interactions, emphasizing the importance of understanding how NPs’ physicochemical properties affect biological systems. It calls for systematic studies to clarify these interactions, highlighting the limitations of non-systematic approaches and advocating for data-driven AI methods, such as ML, to create predictive models.	The article highlights the need for systematic studies to better understand the interactions between NMs and biological systems, pointing out that non-systematic approaches limit insights into the relationships between NP properties and their biological effects. It emphasizes using data-driven AI, such as ML, to create predictive models, which could reduce the need for animal testing. Systematic exploration of NP characteristics—such as size, shape, and surface chemistry—is essential, alongside employing high-speed automation for synthesis and characterization to enhance material exploration. The interactions between NPs and biological systems are complex and warrant multivariate studies to assess combined effects. Effective data management and the development of nanoinformatics tools are critical, as is the use of computational models, such as quantitative structure–activity relationship (QSAR) models, to predict biological properties and design safer NMs. The article also identifies challenges such as the need for improved experimental design and robust datasets while advocating for robotics and advanced ML to advance the field.	Bai et al. [69]
**Nanomedicines and Drug Discovery**		
The document reviews the integration of data curation and ML in advancing nanomedicine development. It discusses the challenges and opportunities in this field and emphasizes the need for collaboration between researchers and data scientists to leverage large datasets for predictive analytics.	Combining data curation with ML enhances the development of nanomedicines by efficiently predicting NM behaviors and optimizing formulations compared to traditional methods. Nanoinformatics integrates computer science, information technology, nanotechnology, and medicine to analyze NM data and accelerate clinical applications. ML algorithms predict the properties of NMs—such as synthesis parameters, efficacy, and toxicity—relying on large, curated datasets for accuracy. Key challenges include the need for unbiased datasets, standardized formats, and collaboration among research groups, which can be addressed with automated systems and advanced data mining. Recent studies highlight ML’s potential to predict drug–excipient interactions and NP stability, showcasing AI’s role in enhancing nanomedicine. Collaboration between AI developers and nanoinformaticians is essential for creating standardized databases that support robust ML analyses. Data-driven approaches, including ML, can streamline nanomedicine processes, reducing the need for extensive trial-and-error experimentation and animal testing. Standardization in data procurement and reporting is crucial for integrating curated data into AI platforms and improving predictive model generalizability.	Chen et al. [34]
The document analyzes systemic delivery barriers of NPs in nanomedicine, introducing “NP blood removal pathways” (NBRP) and strategies to improve NP. It emphasizes the challenges NPs face due to interactions with the body’s blood clearance mechanisms.	The term NP blood removal pathways (NBRP) refers to the mechanisms of NP clearance from the blood, involving both cell-dependent and -independent pathways. Various organs (blood, liver, spleen, lungs, bone marrow, skin, lymph nodes, kidneys, and tumors) significantly influence NP accumulation, affecting their clearance and biodistribution. The physicochemical properties of NPs—size, shape, surface charge, and modifications—are critical to their interactions with NBRP and overall behavior in vivo. For instance, PEGylation can enhance circulation time and reduce liver accumulation. Strategies to enhance NP delivery include Cell uptake saturation: Overloading NBRP with non-therapeutic NPs to prolong the presence of therapeutic ones; Endocytosis inhibition: Using drugs to reduce NP uptake by NBRP cells; Cell depletion: Using drugs to lower macrophage populations, minimizing NP sequestration. Preclinical studies (2011–2021) emphasize the importance of surface chemistry and material composition in NP pharmacokinetics, with Bio-PEG modifications showing the best results. The review notes the need for standardized experimental protocols and data reporting to facilitate comparison across studies. The authors suggest creating a central database for nanobio datasets and utilizing AI to enhance nanomedicine development.	Wang et al. [70]
The document discusses the use of ML in the environmental risk assessment (ERA) of NMs to promote sustainability. It highlights how ML can improve data collection, exposure assessment, hazard identification, and risk characterization within ERA. The article emphasizes the need for clear strategies and standards to integrate ML, ensuring data reliability, transparency, and traceability.	AI tools, particularly ML, are increasingly utilized in ERA, but there are no established standards for their integration, highlighting a significant gap. A critical next step is to develop a workable strategy for implementing AI in ERA, focusing on data foundations, methodologies, and managing uncertainties. High-quality data, adhering to FAIR principles (Findable, Accessible, Interoperable, and Reusable), are essential for effective ERA and ML applications. Challenges include missing data and the risks associated with uncurated or fake data. While ML can estimate missing values, it needs proper training and validation datasets. ML techniques, such as supervised and unsupervised learning, are already applied in ERA, but guidance on their use and criteria is required due to their influence on risk decisions. ML holds promise for hazard identification and enhancing exposure assessment by integrating various data types; however, its complexity can hinder transparency and traceability. A comprehensive strategy for ML in ERA should align with sustainability goals, such as the European Green Deal, and establish controls to ensure the reliability of ML models. The need for further research is emphasized to develop underexplored areas of ML, aiming for responsible usage to protect future generations.	Scott-Fordsmand and Amorim [71]
The document discusses the role of AI and DL in transforming drug discovery and development. It highlights challenges in traditional drug design, such as low efficacy, off-target delivery, high costs, and lengthy timelines. Advances in AI and ML have modernized processes like peptide synthesis, virtual screening, toxicity prediction, and pharmacophore modeling. The text traces the evolution from ML to DL and the integration of big data, covering stages of drug development, including drug screening, QSAR modeling, drug repurposing, and predicting physicochemical properties. It also explores AI’s use in de novo drug design, manufacturing, and clinical trial design, particularly for neurodegenerative diseases, emphasizing AI’s potential to enhance efficiency, reduce costs, and improve accuracy in drug discovery.	Traditional drug design struggles with low efficacy, off-target delivery, high costs, and lengthy timelines. AI and ML, especially DL, have transformed drug discovery, enhancing processes like peptide synthesis, virtual screening, toxicity prediction, drug monitoring, and pharmacophore modeling. The evolution of AI has benefited from advancements in computational power, making it more effective in drug discovery. The integration of big data from genomics and proteomics has improved the efficiency and accuracy of AI applications in drug discovery. Key applications of AI in drug development include Primary and secondary drug screening: AI aids in classifying cells and predicting bioactivity; QSAR modeling: Predicts the relationships between chemical structures and biological activities; Drug repurposing: Identifies new uses for existing drugs, expediting development; Predicting physicochemical properties: Assists in selecting viable drug candidates. AI facilitates the design of novel drug molecules and optimizes clinical trial management, improving success rates and reducing costs. It has proven effective in identifying targets and inhibitors for complex neurodegenerative diseases. Despite its potential, challenges like the need for high-quality data and model reliability persist. Collaboration between pharmaceutical firms and AI developers is essential for progress.	Gupta et al. [72]
The document reviews the role of AI in accelerating drug discovery for G-protein-coupled receptors (GPCRs). It highlights AI’s application at various stages of the drug discovery process, from gaining insights into GPCRs to predicting ligand interactions and clinical outcomes. The review emphasizes AI’s benefits, such as increased speed, efficiency, and cost effectiveness, while also addressing challenges like the need for large datasets and complex models. Lastly, it anticipates future advancements in AI that could further revolutionize GPCR drug discovery, focusing on the importance of open-source data, unsupervised learning, interpretable models, and precision medicine.	Over the last decade, AI and ML have become prominent in the GPCR field, with 3.6% of all GPCR research in 2022 incorporating AI. AI can be utilized in various stages of drug discovery, including classifying GPCRs and their subtypes, predicting mutation impacts on GPCR function, modeling GPCR structures (e.g., AlphaFold2), assessing GPCR–ligand interactions and bioactivity, conducting virtual ligand screening and drug design. ML techniques like support vector machines, decision trees, and DNNs and CNNs have enhanced GPCR drug discovery. AI accelerates the drug discovery process by automating data analysis, improving prediction accuracy, and reducing costs. Challenges include the need for extensive datasets, model complexity, and the interpretability of AI outcomes. Future advancements may lead to increased open-source data access, unsupervised learning, interpretable AI models, a better understanding of GPCRs and diseases, precision medicine approaches, and automated research tools.	Nguyen et al. [73]
The document reviews the role of AI in predicting protein–ligand interactions (PLIs), focusing on its applications in drug discovery.	AI, especially ML and DL, greatly improves the prediction of PLIs, essential for drug discovery. Key databases like PDBBind, LigASite, BioLiP, and BindingDB support training and validation of AI models for PLI prediction. Prediction methods: Binding site prediction: Classical ML techniques (SVM, RF) are effective, but recent DL approaches (CNNs) show improved accuracy; Binding affinity prediction: ML methods (RF, ensemble) and advanced DL techniques (3D CNNs, hybrid models) provide better predictions; Binding pose prediction: ML and DL methods evaluate docking results, with recent progress in reinforcement learning optimizing ligand poses. Challenges in binding site prediction include imbalanced datasets. Integrating binding site, affinity, and pose predictions into a single model through multitask learning is a promising research direction, with advanced DL architectures, especially those using attention mechanisms, likely to enhance accuracy further.	Dhakal et al. [6]
**Protein–Protein Interactions**		
The document reviews ML solutions for predicting PPIs, emphasizing the importance of proteins in biological processes and biomolecular condensates. It highlights the role of ML, especially DL, in PPI prediction and the need for high-quality training data and effective data representation. The review covers various ML methodologies, including traditional techniques like support vector machines (SVMs) and random forests (RFs), as well as DL approaches like CNNs and GCNs. It also outlines challenges in PPI prediction, such as false positives and the need for more comprehensive datasets.	Proteins are vital for biological processes, with their interactions essential for cellular functions. Understanding PPIs provides insights into cellular mechanisms and diseases. Proteins can form biomolecular condensates, which are influenced by the cell’s needs and play various roles. ML, particularly DL, shows promise in predicting PPIs, utilizing methods like SVMs, RFs, CNNs, and GCNs. High-quality training data and effective representation are key to ML success, highlighting the need for comprehensive datasets. Despite advancements, current ML approaches for PPI prediction face limitations, including issues with accuracy and complexity. False positives in predictions are a significant concern, necessitating rigorous validation and benchmarking. Innovations like AlphaFold2 enhance protein structure predictions, benefiting PPI methods, but gaps remain in their performance. More high-resolution examples of protein complexes are needed, and distinguishing between functional and non-functional interactions is an ongoing challenge.	Casadio et al. [2]
The article explores the use of AI to characterize PP binding interfaces, aiming to improve the assembly of protein complexes and enhance the predictions of PPIs based on their structural properties.	The study revealed that PP binding interfaces are highly similar and can be captured by AI. By dividing these interfaces into interacting fragment pairs and utilizing a generative model, researchers encoded them into a low-dimensional latent space, enabling the generation of new conformations. An autoencoder neural network accurately reconstructed these fragment pairs, with deviations under 1 A from native positions. Clustering in the latent space using a self-organizing map (SOM) facilitated the visualization of these correlations. The AI-generated fragment pairs closely resembled native structures, aiding in the assembly of protein complexes. The research highlights the degenerate nature of conformational space at PP binding interfaces, suggesting that a limited number of fragment pairs can represent diverse interactions. This method could aid in predicting unknown PPIs, enhancing our understanding of biological processes and therapeutic development.	Su et al. [19]
The document outlines a sequence-based ML method, PhosPPI, for predicting how phosphorylation affects PPIs and identifies functional phosphorylation sites influencing PPI. It also discusses the role of phosphorylation in diseases like cancer and Alzheimer’s, emphasizing the need for computational methods due to the labor-intensive and costly nature of traditional experimental techniques.	PhosPPI is a sequence-based ML method developed to predict the effects of phosphorylation on PPIs. It includes two models: PhosPPI-1 for identifying functional phosphorylation sites and PhosPPI-2 for assessing the impact of phosphorylation on PPI. Outperforming methods like Betts, HawkDock, and FoldX in accuracy and AUC, PhosPPI does not require 3D protein structures, enhancing its applicability. A user-friendly web server allows input of protein sequences and phosphorylation sites for predictions (https://phosppi.sjtu.edu.cn/ (accessed on 10 December 2024). Distinct sequence patterns characterize functional phosphorylation sites, with specific amino acids enriched nearby. PhosPPI conducted large-scale predictions on known PPIs, showing that phosphorylation effects are consistent across homo and heterocomplexes. A case study on integrin and filamin highlighted PhosPPI’s ability to identify novel regulatory phosphorylation sites, potentially advancing the understanding of disease mechanisms and aiding drug development. PhosPPI can help researchers dissect molecular disease mechanisms, understand drug resistance, and develop new therapeutics targeting functional phosphorylation sites.	Hong et al. [74]
The document reviews advancements in ML and DL techniques for predicting PepPIs and PPIs using sequence information. These predictions are crucial for understanding disease mechanisms and drug development. It examines relevant databases, data formats, and feature representations, categorizing ML and DL methods while analyzing their pros and cons. Additionally, it discusses the validation protocols and evaluation metrics for assessing model performance.	Peptides and proteins are vital for biological processes and drug discovery due to their interactions. Traditional methods for detecting PPIs and PepPIs are often time-consuming and costly, leading to false positives. ML and DL have improved the prediction of these interactions, effectively handling large datasets and complex biological data. Databases like BioGRID, HPRD, UniProt, DIP, and STRING offer valuable data for PPI and PepPI predictions. The review categorizes ML and DL methods into tree-based, kernel-based, and NN-based approaches, discussing their pros and cons. Validation protocols and metrics, such as MCC, F1-score, AUC, and PR-AUC, assess model performance. Several web tools, including ScanNet, HHblits, SeqVec, DockThor, HDOCK, HawkDock, and AlphaFold2, enhance predictions. Ongoing challenges include accurate protein structure prediction, managing dynamic interactions, and generalization across species, with future research likely focusing on combining MD with sequence-based predictions.	Ye et al. [75]
The document discusses the importance of explainable AI in predicting PPIs using knowledge-graph-based semantic similarity. It introduces KGsim2vec, a novel approach designed to address the limitations of traditional ML models in providing explainability for these predictions.	KGsim2vec enhances explainability and predictive performance in PPI predictions compared to traditional black-box methods, such as knowledge graph embeddings and GNNs. By computing semantic similarity within a knowledge graph, it offers detailed and interpretable explanations for interactions. The method employs various ML models, such as decision trees and random forest, and generally outperforms black-box techniques. KGsim2vec produces explanations that reflect biological phenomena and reveal data biases, achieving a good balance between size and informativeness. Frequent decision tree rules align with existing biological knowledge, reinforcing the model’s validity and interpretability. Filtering out less informative semantic aspects minimally impacts performance but can affect explanation informativeness, highlighting the method’s robustness.	Sousa et al. [76]
This document reviews recent advancements in DL techniques for analyzing PPIs from 2021 to 2023. It highlights the impact of DL methods in computational biology, particularly in understanding the PPIs crucial for various biological functions and therapies.	Various DL approaches, including GNNs, CNNs, RNNs, autoencoders, attention mechanisms, transformers, multitask and multimodal learning, and transfer learning, have all been utilized effectively for PPI prediction. Each method has unique strengths for handling different PPI data characteristics. Key models: AlphaFold: Predicts protein structures with high accuracy, significantly influencing PPI prediction; GNNs: Model the graph-like structure of PI networks effectively; CNNs: Capture local dependencies and biological feature hierarchies in protein sequences; RNNs: Handle sequential data, with LSTM networks managing long-term dependencies well. DL models rely on robust, balanced datasets, but biological datasets are often imbalanced and noisy. Combining diverse data types (sequence, structural, functional) presents integration challenges. While complex models enhance performance, they often reduce interpretability and biological insight extraction. Developing advanced data augmentation and robust regularization methods to improve data quality. Exploring better feature extraction and fusion techniques for enhanced learning efficiency. Improving model interpretability using attention mechanisms and explainable AI. Expanding applications to drug discovery, personalized medicine, and environmental genomics are the suggested future directions. Emerging topics include focusing on predicting binding sites, residue–residue interactions, and protein association rates; utilizing hybrid models to blend different learning techniques for optimal performance; leveraging models like AlphaFold for advanced PPI prediction and protein complex modeling.	Lee [77]
The document highlights the use of ML in studying PPIs, focusing on uromodulin (UMOD) activation and polymerization related to egg zona pellucida (ZP) filaments. It examines ML techniques like AlphaFold2 and ColabFold for predicting protein structures and interactions. The findings suggest that these tools can effectively model conformational changes and interactions in protein polymerization, even without prior structural knowledge, demonstrating the potential of ML to elucidate complex biological processes.	AlphaFold2 accurately modeled the structure of the polymerization-inhibited UMOD ZP module, aligning with experimental data. For the polymerization-activated state, it predicted conformational changes that reflect key interactions in the cryo-EM structure of the UMOD filament. Using ColabFold, the study successfully modeled interactions between activated UMOD subunits, mirroring the main interactions in the experimental filament structure. The modeling approach was also extended to egg coat proteins ZP2 and ZP3, suggesting their interdomain linkers adopt conformations that promote polymerization, akin to UMOD. These findings highlight the capability of tools like AlphaFold2 and ColabFold to predict individual protein structures as well as complex interactions and conformational changes in polymerization.	Jovine [78]
The document reviews protein–DNA/RNA interactions using machine intelligence tools, focusing on computational methods for predicting these interactions. It discusses the evolution from traditional ML to DL, outlining the strengths and weaknesses of each approach. The review also covers strategies for digitizing biological sequences and their applications in studying protein–DNA/RNA interactions.	The review outlines the shift from traditional ML methods, such as SVM and random forests, to DL techniques, such as CNN and RNN, for predicting protein–DNA/RNA interactions. While structure-based approaches usually outperform sequence-based ones, the latter are more common when structural data are lacking. ML methods are categorized into supervised, unsupervised, semi-supervised, and reinforcement learning, each suited for different data types. Key to these models is sequence digitization, with traditional methods relying on feature extraction and DL using sequence representation learning techniques like one-hot encoding and transformer models like BERT. The review discusses various tools for identifying binding proteins and predicting binding sites, noting a trend toward DL algorithms. Challenges such as the lack of interpretability in DL are highlighted, with future research suggested in developing interpretable models and integrating structural information from databases like AlphaFold. The review emphasizes the transformative role of AI and big data in enhancing computational methods in bioinformatics, providing an overview of current trends and future directions in the field.	Cui et al. [64]

**Table 4:** Comparison of ML training models.

Model	Description	Advantages	Limitations
Random Forests (RFs)	RFs are ensemble learning methods that create multiple decision trees during training and output the mode of the classes (classification) or mean prediction (regression) of the individual trees.	Robustness, versatility, and feature importance	Computationally intensive, less interpretable
Neural Networks (NNs)	NNs consist of layers of interconnected nodes (neurons) that process input data to predict outputs. They can be shallow (few layers) or deep (many layers).	Flexibility, scalability	Long training time, prone to overfitting, less interpretable
Support Vector Machines (SVMs)	SVMs are supervised learning models that find the optimal hyperplane to separate data into different classes. They can handle linear and non-linear classification using kernel functions.	Effective in high-dimensional spaces, flexible with kernels	Not suitable for large datasets; requires careful parameter tuning
Extreme Gradient Boosting (XGBoost)	XGBoost is an optimized gradient boosting algorithm that builds an ensemble of decision trees sequentially, where each tree corrects the errors of the previous ones.	High accuracy, fast training, regularization to prevent overfitting	Complex implementation, resource-intensive

**Table 5:** Key databases on protein interactions and their applications.

Database	Description	Applications	URL
IntAct Molecular Interaction Database	A freely accessible, open-source database that provides molecular interaction data curated from the scientific literature.	Researchers can use IntAct to obtain high-quality, experimentally validated PPI data for training ML models. It is particularly useful for creating training sets for supervised learning algorithms.	https://www.ebi.ac.uk/intact (accessed on 8 March 2025).
Biological General Repository for Interaction Datasets (BioGRID)	A comprehensive database that archives and disseminates genetic and protein interaction data, including chemical interactions, from model organisms and humans.	Researchers can use BioGRID to construct PPI networks for different organisms, which can be used to identify essential proteins and study disease mechanisms. The extensive interaction data can be used to train machine-learning models for predicting new PPIs.	https://thebiogrid.org/ (accessed on 8 March 2025).
Search Tool for the Retrieval of Interacting Genes/Proteins (STRING)	A database of known and predicted PPIs, including functional associations derived from various sources, such as genomic context, high-throughput experiments, co-expression, and text mining.	STRING can be used to analyze functional networks and identify key proteins involved in specific biological processes. The predicted interactions in STRING can be used to augment training datasets for ML models, especially when experimental data are limited.	https://string-db.org/ (accessed on 8 March 2025).
Protein Data Bank (PDB)	A repository for the 3D structural data of large biological molecules, such as proteins and nucleic acids.	PDB is essential for researchers focusing on structure-based PPI predictions. It provides atomic-resolution structures that can extract features for ML models.	www.wwpdb.org (accessed on 8 March 2025). http://www.rcsb.org (accessed on 8 March 2025).
PDBbind	A comprehensive collection of experimentally measured binding affinity data for biomolecular complexes deposited in the PDB.	Researchers can use PDBbind to obtain binding affinity data, which are crucial for training models predicting PPIs’ strength.	https://www.pdbbind-plus.org.cn/ (accessed on 8 March 2025).
Structural Kinetic and Energetic Database of Mutant Protein Interactions (SKEMPI)	Contains data on changes in thermodynamic parameters and kinetic rate constants upon mutation for PPIs.	SKEMPI is useful for studying the effects of mutations on PPIs and for training ML models to predict these effects.	https://life.bsc.es/pid/skempi2 (accessed on 8 March 2025).
Human Protein Reference Database (HPRD)	A protein-centric database that provides information on human protein interactions, including the relationships between proteins and diseases; it covers over 30,000 human proteins.	HPRD can be used to study the relationships between proteins and various diseases, aiding in identifying potential therapeutic targets. The curated interaction data can serve as positive samples for training ML models to predict PPIs.	http://www.hprd.org/ https://www.hsls.pitt.edu/obrc/index.php?page=URL1055173331 (accessed on 8 March 2025).
Universal Protein Resource (UniProt)	A comprehensive resource for protein sequence and functional information, including reviewed entries in Swiss-Prot and unreviewed entries in TrEMBL, as well as 3D structural data.	Researchers can extract detailed protein information, including sequence, structure, and function, for use as features in ML models. The high-quality, curated data in UniProt can be used to train and validate models for predicting PPIs and PepPIs.	https://www.uniprot.org/ (accessed on 8 March 2025).
Database of Interacting Proteins (DIP)	A curated database of experimentally determined PPIs, including interactions from various organisms; it provides a gold standard dataset for PPI studies.	DIP can benchmark the performance of ML models by providing a reliable set of positive and negative interaction samples. The interaction data can train models for predicting PPIs, especially in yeast and other model organisms.	http://dip.doe-mbi.ucla.edu/ (accessed on 9 March 2025).
GPCR–Ligand Association (GLASS)	A comprehensive database that contains experimentally validated GPCR–ligand associations.	It is used to train models for predicting GPCR–ligand interactions, which is critical for drug discovery and repurposing programs. The database provides a wealth of information on known GPCR–ligand pairings, helping to identify potential drug candidates.	https://zhanggroup.org/GLASS/ (accessed on 9 March 2025).
BindingDB	A public, web-accessible database of measured binding affinities, focusing on the interactions of proteins considered to be drug targets with small, drug-like molecules.	Researchers use BindingDB to train ML models to predict binding affinities and perform virtual screening of potential drug candidates. It is particularly useful for understanding the strength of interactions between ligands and their target proteins.	http://www.bindingdb.org (accessed on 9 March 2025).
Drug and Drug Target Database (DrugBank)	A comprehensive resource that combines detailed drug data with comprehensive drug target information.	DrugBank is used for repurposing, understanding drug mechanisms, and predicting off-target effects. It provides a rich dataset for training ML models to predict drug–target interactions and explore drug pharmacological properties.	https://www.drugbank.com/ (accessed on 9 March 2025).
A Chemogenomic Database (ChEMBL)	A large-scale bioactivity database containing information on small molecules’ bioactivity and their drug-like properties.	ChEMBL is widely used to train ML models in virtual screening, bioactivity prediction, and de novo drug design. It provides extensive data on the biological activities of compounds, which are essential for developing predictive models.	https://www.ebi.ac.uk/chembl/ (accessed on 9 March 2025).
A public chemical information resource (PubChem)	A free chemistry database maintained by the National Center for Biotechnology Information (NCBI), containing deep-learning information on the biological activities of small molecules.	PubChem is used for chemical informatics research, including training ML models to predict chemical properties, bioactivity, and toxicity. It is a valuable resource for researchers exploring the chemical space and identifying potential drug candidates.	http://pubchem.ncbi.nlm.nih.gov/ (accessed on 9 March 2025).
G-protein-coupled receptor database (GPCRdb)	A database dedicated to G-protein-coupled receptors, providing information on receptor sequences, structures, and functions.	GPCRdb is used for structural modeling, understanding receptor–ligand interactions, and exploring receptor functions. It supports the development of ML models for predicting GPCR activity and designing receptor-specific drugs.	https://gpcrdb.org/ (accessed on 9 March 2025).

**Table 6:** Opportunities, limitations, and challenges in the application of AI and ML techniques for characterizing protein corona, nanobio interactions, nanomedicines and drug discovery, and protein–protein interactions.

Topic	Opportunities	Limitations and Challenges
*Nanotoxicology and nanomaterial research*	Develop and refine ML algorithms to predict NM interactions with cells more accurately. Establish standardized protocols for characterizing and reporting NM interactions with biological systems. Expand research to include a wider variety of cell types and biological models. Promote interdisciplinary collaboration among material scientists, biologists, toxicologists, and computational experts.	Complex interactions between NMs and biological systems make accurate prediction of behavior and toxicity challenging. Lack of comprehensive studies explaining the influence of NM properties on cell CSI and NAFs. Inherent heterogeneity in NM properties complicates the assessment of cellular fate and toxicity. Absence of standardized protocols for characterizing and reporting NM interactions with cells. Specialized equipment and expertise required for advanced imaging and analytical techniques may not be readily available. Variability in biological responses to NM exposure adds complexity to toxicity assessment. Long-term effects of chronic exposure to NMs are not well understood.
*Protein corona prediction*	Extract additional features related to proteins to enrich feature representation. Develop models that generalize across multiple proteins. Explore sophisticated neural network architectures to avoid overfitting. Create integrated models for both classification and regression tasks. Focus on interpretable ML methods to understand decision-making processes.	Modeling individual proteins separately limits generalization ability. High duplication in datasets and small datasets for some proteins lead to undertraining and biased predictions. Complex neural network architectures tend to suffer from overfitting. Current approaches do not fuse features of proteins during model training. Interpretable analysis for numerous baseline models is tedious and complex. Some regression models exhibit suboptimal performance, indicating non-optimal datasets.
*Nanomedicine and drug discovery*	Leverage ML to design safer and more effective NPs for applications in nanomedicine, biosensing, and organ targeting. Collect and incorporate diverse and comprehensive datasets to improve model robustness. Conduct real-world validation of ML models through experimental studies and clinical trials. Combine data from various omics fields for a comprehensive understanding of NM interactions.	Lack of large, high-quality, and unbiased datasets for training robust ML models. Non-standardized reporting metrics and varying data formats hinder data integration. Manual data curation is time-consuming and low-throughput. ML models can easily overfit training data, reducing generalization ability. Heterogeneity and complexity of NMs make accurate behavior prediction challenging. Fragmented and inaccessible NM databases limit data sharing. Bridging the gap between data scientists, nanotechnologists, and biomedical researchers is challenging. High computational costs and resource requirements for detailed MD simulations.
*Protein function prediction*	Develop sophisticated DL and AI methods to integrate multiple modalities of input data. Utilize evolutionary information from protein sequences to improve predictions. Create LLMPs that can be fine-tuned for function prediction. Foster collaboration among ML, AI, and bioinformatics communities.	Integrating multiple modalities of input data to improve prediction accuracy is challenging. Effectively utilizing evolutionary information from protein sequences remains difficult. Improving prediction accuracy for rare or novel GO terms is a challenge. Increased model complexity and scalability issues arise when integrating multiple data modalities. Curating high-quality training and test datasets is essential but challenging.
*Nanobio interactions and nanoinformatics*	Systematically explore physicochemical properties of NPs and their interactions with biological systems. Use combinatorial chemistry and high-throughput methods to generate large datasets. Develop advanced computational models to predict biological effects of NPs. Improve data management and sharing protocols.	Understanding interactions between NPs and biological systems is highly complex. Non-systematic studies and limited scope hinder understanding of combined effects of multiple properties. Lack of reliable and comprehensive datasets on nanobio interactions. Differences in preparation methods, cell types, and experimental conditions complicate comparisons. Large surface-to-volume ratio of NPs means surface modifications significantly influence biological effects. NPs undergo various transformations in biological environments, adding complexity. Lack of universally accepted data formats and protocols limits data sharing.
*Environmental risk assessment*	Incorporate ML into ERA to improve data gathering, exposure assessment, hazard identification, and risk characterization. Develop comprehensive strategies for implementing ML in ERA. Combine ML with the IoT for real-time environmental monitoring and management.	No agreed-upon standards or strategies for incorporating ML in ERA. High-quality and comprehensive data are crucial but often incomplete or uncurated. ML models can inherit biases and historical errors from training data. Complexity and “black box” nature of ML models make them difficult to interpret. Overfitting and underfitting issues affect model generalizability and accuracy. No studies have integrated ML across all steps of ERA. Ensuring reproducibility of ML models is challenging.
*Drug discovery and development*	○ Use AI to improve virtual screening, toxicity prediction, and QSAR modeling. ○ Enhance personalized medicine by analyzing patient-specific data. ○ Integrate genomics, proteomics, and other omics data through AI for a comprehensive understanding of disease mechanisms. ○ Facilitate drug repurposing and optimize clinical trials using AI.	High-quality, well-curated data are essential but often scarce and inconsistent. Handling sensitive patient data raises privacy and security concerns. Lack of interpretability in AI models hinders understanding and regulatory approval. Integrating AI into traditional workflows requires significant changes in infrastructure and processes. Regulatory bodies require extensive validation and transparency. Substantial computational power and resources are needed for AI models. AI models can inherit biases from training data. Ethical questions arise regarding data privacy, transparency, and job displacement.
*Protein–protein interactions*	○ Develop more effective methods for representing protein sequences and structures. ○ Enhance computational efficiency of ML models for broader accessibility. ○ Move beyond interaction prediction to understand functional and contextual relevance of PPIs. ○ Integrate sequence features, docking scoring functions, and protein binding site predictions into AI models.	High-quality, reproducible experimental data are essential but often inconsistent. Properly representing complex protein sequences and structures is challenging. ML models can generate false positives, predicting interactions that do not occur in reality. Limited high-resolution protein complexes available for training. Dynamic and context-dependent nature of PPIs adds complexity. Significant computational resources required for training deep-learning models. Rigorous benchmarking and validation procedures are necessary but challenging.
*Explainable AI*	○ Improve the explainability of AI models to ensure reliability and interpretability. ○ Use ontologies and knowledge graphs to enhance model explainability. ○ Develop scalable methods to handle large datasets and complex knowledge graphs efficiently.	Biomedical domain involves highly complex data, making model development challenging. Many AI models are “black boxes” and lack interpretability. Knowledge graphs often lack meaningful interpretations specific to the biomedical domain. Semantic similarity scores are often too compact, reducing complex relationships to single numerical scores. AI models can be influenced by biases in the data. Trade-off between model performance and explainability. Evaluating the quality of explanations is challenging.
*Deep learning for PPI analysis*	○ Integrate multimodal data sources and diverse biological features to improve prediction accuracy. ○ Develop techniques to enhance model transparency and interpretability. ○ Apply DL models to interdisciplinary domains like drug discovery and personalized medicine.	Imbalanced class distributions and noise in biological datasets lead to biased predictions. Managing and harmonizing heterogeneous data types is complex. DL models can be highly complex, compromising interpretability. Accurately predicting the structures of PP complexes remains challenging. GNNs show promise but require further research to address limitations. Fold-and-dock algorithms need continued development and refinement.

## Abbreviations

The following abbreviations are used in this manuscript:

AI	Artificial Intelligence
BindingDB	Binding Database
BioGRID	Biological General Repository for Interaction Datasets
BioLiP	Biologically Relevant Ligand–Protein Binding Interactions Database
BSA	Bovine Serum Albumin
CAFA	Critical Assessment of Protein Function Annotation
ChEMBL	Chemogenomic Database
CNN	Convolutional Neural Network
CNTs	Carbon Nanotubes
CSI	Cell Shape Index
DFT	Density Functional Theory
DIP	Database of Interacting Proteins
DL	Deep Learning
DNA	Deoxyribonucleic Acid
DNNs	Deep Neural Networks
DrugBank	Drug and Drug Target Database
DWCNTs	Doublewalled Carbon Nanotubes
ENMs	Engineered Nanomaterials
ERA	Environmental Risk Assessment
ERT	Extremely Randomized Tree
FAIR	Findable, Accessible, Interoperable, and Reusable
GBDT	Gradient-Boosting Decision Tree
GLASS	GPCR–Ligand Association
GO	Gene Ontology
GPCR	G-Protein-Coupled Receptor
GPCRdb	G-Protein-Coupled Receptor Database
His	Histidine
HPRD	Human Protein Reference Database
IntAct	Molecular Interaction Database
KGsim2vec	Knowledge-Graph-Based Method
KNN	K-Nearest Neighbor
LigAsite	Ligand Attachment Site Database
LightGBM	Light Gradient-Boosting Machine
LLMPs	Large Language Models for Proteins
MD	Molecular Dynamics
ML	Machine Learning
MWCNTs	Multiwalled Carbon Nanotubes
NAFs	Nuclear Area Factors
NBRP	NP Blood Removal Pathways
NMs	Nanomaterials
NN	Neural Networks
NPs	Nanoparticles
RPA	Relative Protein Abundance
PCRO-RLRM	Polypeptide Chemical Reaction Optimized Resistant Logistic Regression Model
PDB	Protein Data Bank
PDBbind	Binding Affinity Data for Biomolecular Complexes Deposited in the PDB
PepPIs/PPIs	Peptide/Protein–Protein Interactions
Phe	Phenylalanine
Phos	Phosphorylation
PLIs	Protein–Ligand Interactions
PSSM	Position-Specific Scoring Matrix
PPIs	Protein–Protein Interactions
QSAR	Quantitative Structure–Activity Relationship
PubChem	Public Chemical Information Resource
R^2^	Coefficient of Determination
RF	Random Forest
RNA	Ribonucleic Acid
SHAP	SHapley Additive exPlanations
SKEMPI	Structural Kinetic and Energetic Database of Mutant Protein Interactions
SOM	Self-Organizing Map
STRING	Search Tool for the Retrieval of Interacting Genes/Proteins
SVM	Support Vector Machine
Trp	Tryptophan
Tyr	Tyrosine
UMOD	Uromodulin
UniProt	Universal Protein Resource
XGBoost	Extreme Gradient Boosting
